# Scoping review of interventions to improve continuity of postdischarge care for newborns in LMICs

**DOI:** 10.1136/bmjgh-2023-012894

**Published:** 2024-01-10

**Authors:** Gulraj Grewal, Sebastian S Fuller, Asma Rababeh, Michuki Maina, Mike English, Chris Paton, Chrysanthi Papoutsi

**Affiliations:** 1 Nuffield Department of Medicine, Center for Tropical Medicine and Global Health, University of Oxford, Oxford, UK; 2 Health Services Unit, KEMRI - Wellcome Trust Research Institute, Nairobi, Kenya; 3 Department of Information Science, University of Otago, Dunedin, New Zealand; 4 Nuffield Department of Primary Care Health Sciences, University of Oxford, Radcliffe Observatory Quarter, Oxford, UK

**Keywords:** Child health, Health systems, Paediatrics, Public Health, Review

## Abstract

**Introduction:**

Neonatal mortality remains significant in low-income and middle-income countries (LMICs) with in-hospital mortality rates similar to those following discharge from healthcare facilities. Care continuity interventions have been suggested as a way of reducing postdischarge mortality by better linking care between facilities and communities. This scoping review aims to map and describe interventions used in LMICs to improve care continuity for newborns after discharge and examine assumptions underpinning the design and delivery of continuity.

**Methods:**

We searched seven databases (MEDLINE, CINAHL, Scopus, Web of Science, EMBASE, Cochrane library and (Ovid) Global health). Publications with primary data on interventions focused on continuity of care for newborns in LMICs were included. Extracted data included year of publication, study location, study design and type of intervention. Drawing on relevant theoretical frameworks and classifications, we assessed the extent to which interventions adopted participatory methods and how they attempted to establish continuity.

**Results:**

A total of 65 papers were included in this review; 28 core articles with rich descriptions were prioritised for more in-depth analysis. Most articles adopted quantitative designs. Interventions focused on improving continuity and flow of information via education sessions led by community health workers during home visits. Extending previous frameworks, our findings highlight the importance of interpersonal continuity in LMICs where communication and relationships between family members, healthcare workers and members of the wider community play a vital role in creating support systems for postdischarge care. Only a small proportion of studies focused on high-risk babies. Some studies used participatory methods, although often without meaningful engagement in problem definition and intervention implementation.

**Conclusion:**

Efforts to reduce neonatal mortality and morbidity should draw across multiple continuity logics (informational, relational, interpersonal and managerial) to strengthen care after hospital discharge in LMIC settings and further focus on high-risk neonates, as they often have the worst outcomes.

**WHAT IS ALREADY KNOWN ON THIS TOPIC**

- Mortality rates for newborns in low-income and middle-income countries are a growing concern, with most deaths occurring within 30 days postdischarge.
- Continuity of care approaches have gained interest as a way of reducing neonatal mortality rates and achieving Sustainable Development Goal 3.
- Previous scoping reviews have focused primarily on continuity of care in high-income countries.

**WHAT THIS STUDY ADDS**

- Continuity of information provision was mostly attempted via home visits by community health workers (CHWs).
- Home visits were easier to implement in contexts where CHWs were well established and trusted within the community.
- Continuity of interpersonal relationships emerged as a salient consideration, highlighting the importance of relationships between family members, between healthcare workers or between people within the community.

**HOW THIS STUDY MIGHT AFFECT RESEARCH, PRACTICE OR POLICY**

- Establishing CHW programmes within communities, although challenging, may aid community-based care.
- Interventions face challenges with implementation in contexts with patriarchal gender norms, hence more effort may be required for community engagement or interventions should be designed to include men.
- Future research should focus specifically on high-risk newborns with interventions drawing across multiple continuity logics.

## Introduction

The newborn period (the first 28 days) of a child’s life is when they are most vulnerable, with 2.4 million newborn deaths occurring globally in 2020.^1^ Nearly two-thirds of newborn deaths in developing countries occur within the first 3 days of life.^2^ A disproportionate burden is found in sub-Saharan Africa (43% of newborn deaths in 2020).^1^ Sustainable Development Goal 3 (SDG 3) aims for all countries to have a neonatal mortality rate (NMR) at least as low as 12 per 1000 live births.^3 4^ Between 1990 and 2017, the global NMR dropped from 36.6 deaths per 1000 to 18.0 deaths per 1000. However, improvements need to be accelerated in areas with persistently high NMR, such as sub-Saharan Africa.^5^

The WHO recommends at least four postnatal care visits in the first 6 weeks postdelivery for all babies, which can include home visits.^6^ The WHO also recommends that postnatal visits should include breastfeeding support and advice, assessment of danger signs, cord care and mental health support for mothers to create positive postnatal experiences.^6^ Yet previous research in low-income and middle-income countries (LMICs) has identified high rates of discontinuation in postnatal care, in contrast to antenatal care.^7–9^ Postnatal visits can be affected by income level, distance from facilities and mode of delivery.^8^ Addressing these barriers is critical, especially for sick newborns as postdischarge mortality and morbidity is a growing concern,^10^ with most postdischarge deaths occurring within the first 30 days postdischarge.^11^ Robust follow-up systems have been highlighted as a necessity in LMICs for sick newborns.^12^ Continuity of care has recently been suggested as a way of reducing neonatal mortality for this group by linking pregnancy, delivery and postnatal care, as well as strengthening communication between households and hospitals,^13 14^ which can lead to achievement of the SDG targets. Continuity of care is defined as ‘the provision of coordinated care and services over time and across levels and disciplines, which is coherent with the patient’s health needs and personal circumstances’.^15^ There are different approaches to care continuity, each underpinned by different assumptions or logics about how continuity might be achieved, and the levels of care and how the extent to which care is provided may differ over time. In the context of child health continuity, this has been characterised as involving time (the period from prepregnancy to childhood) and space (care from facilities to community-based care).^16^

Continuity can have a positive effect on maternal and child healthcare (MCH), including improving breastfeeding initiation and duration, and outcomes related to maternal and newborn health status, such as improved maternal mental health.^17^ Several interventions have been implemented to improve continuity, for example, MCH booklets introduced in Angola.^18^ Previous reviews on MCH in high-income countries have also emphasised the importance of strengthening continuity, highlighting this as a priority area for research in LMICs.^19^

Participatory approaches by engaging with the people who will ultimately interact with the intervention have drawn interest in recent years. These approaches aim to form collaborations with stakeholders and participants as opposed to viewing them as research subjects.^20^ Participatory approaches may lead to improved care continuity. For example, one review found using participatory methods to redesign forms with healthcare workers led to improved documentation.^21^ While participatory approaches hold promise, it is worth noting that few published studies mention power and control, which can hinder the egalitarian principles participatory approaches are based on.^22^

This scoping review aims to map and describe interventions and approaches that have been used in LMICs to improve care continuity for newborns, with a focus on the period after discharge from healthcare facilities.

### Research questions

We used the following research questions to guide the review:

1. What interventions, innovations or support mechanisms have been proposed to support continuity in postdischarge care for newborns in LMICs?
2. To what extent have participatory methods been used to develop these interventions?
3. What are the different logics (premises or assumptions) that underpin interventions to establish continuity?
4. What opportunities and challenges for improving continuity of newborn care within the period after birth have been identified in previous studies?

## Methods

We used the five-step scoping review method outlined by Arksey and O’Malley,^23^ which included setting the research questions; identifying relevant studies; study selection; data charting; collating, summarising and reporting the results. The first author (GG) incorporated the updates by Peters *et al*,^24^ hence the population, concept, context (PCC) framework was used to guide the inclusion and exclusion criteria.

### Search strategy

Initially GG conducted exploratory searches to gain familiarity with the literature, define key terms and develop the search strategy. Search terms and information sources were selected based on keywords and databases used in previous reviews in similar topic areas.^15 25–27^ Two librarians reviewed the search strategy to confirm all relevant terms had been included. The full search terms can be found in online supplemental appendix 1. GG searched seven databases: MEDLINE, CINAHL, Scopus, Web of Science, EMBASE, Cochrane library and (Ovid) Global health. The search was limited to papers published in English between 2001 and 2021.

10.1136/bmjgh-2023-012894.supp1 Supplementary data

#### Eligibility criteria

GG screened the titles and abstracts of retrieved articles using Rayyan. One of the coauthors (AR) reviewed a random 10% sample of articles to refine the process of article inclusion and exclusion; disagreements in 26 of the articles reviewed were resolved through discussion with reference to the inclusion and exclusion criteria. Subsequently GG retrieved and read the full texts of all articles remaining after title and abstract screening to assess them for inclusion. Articles had to meet the PCC criteria: the population under investigation was newborn babies (defined as babies under 28 days of age),^28^ the concept was defined as continuity of care after discharge and the context was LMICs. There were no limits on the study design if primary data were used. Articles were only included if there was a clearly defined intervention to improve continuity of care for newborns in LMICs.

### Data charting and synthesis

GG extracted descriptive information for each of the articles included in the review using an Excel spreadsheet (see table 1, online supplemental appendix 2). A second reviewer (AR) charted data from a random 10% sample of articles to get a second opinion on data abstraction; differences were resolved through discussion. The table provides an overview of study characteristics such as author, title, year of publication, study location, study design, aim, type of intervention, methods and sample size.

**Table 1:** General characteristics of interventions included in the review

Title of paper	Author	Intervention	Aim	Who implemented the intervention?	Target of intervention	Purpose/focus of intervention	Continuity logic
Intermittent kangaroo mother care and the practice of breastfeeding late preterm infants: results from four hospitals in different provinces of China	Zhang *et al*	HCW training	Behaviour change	HCWs in hospitals	Mothers of late preterm babies	KMC and BF	Informational
Breast crawl at birth, effect on breastfeeding rate and infant growth in infants delivered at an urban tertiary care public hospital: A randomized controlled trial	Mulupuru *et al*	Breast crawl (immediate skin to skin contact until breastfeeding (BF))	Behaviour change	HCWs in hospitals	Mothers of term babies	BF	Interpersonal
Lower mortality is observed among low birth weight young infants who have received home-based care by female community health volunteers in rural Nepal	Neupane *et al*	Home visits by CHWs	NMR	CHWs	Mothers of LBW babies	Community-based newborn care, including administering antibiotics and referrals.	Relational
Effect of community-based newborn-care intervention package implemented through two service-delivery strategies in Sylhet district, Bangladesh: a cluster-randomised controlled trial.	Baqui *et al*	Home visits by CHWs	NMR	CHWs	Married women of reproductive age (15-49) (community-based care). Pregnant women (home-based care)	Community-based newborn care, including community mobilisation, administering antibiotics and referrals.	Informational, Management, Interpersonal
Process evaluation of a knowledge translation intervention using facilitation of local stakeholder groups to improve neonatal survival in the Quang Ninh province, Vietnam.	Eriksson *et al*	Group-based activities	Process	Trained facilitators from the Women’s Union	Local healthcare staff and local key stakeholders (trained professionals, influential commune members).	Identify local problems and solutions	Interpersonal
Low birth weight and preterm neonates: can they be managed at home by mother and a trained village health worker?	Bang *et al*	Home visits by CHWs	NMR	CHWs	Mothers of babies who are preterm or LBW.	Health education, management of newborns and referrals to care.	Informational, Relational
Effects of quality improvement in health facilities and community mobilization through women’s groups on maternal, neonatal and perinatal mortality in three districts of Malawi: maiKhanda, a cluster randomized controlled effectiveness trial	Colbourn *et al*	Group-based activities	NMR	Volunteer facilitators	Pregnant women	Identify local problems and solutions	Informational, Management, Interpersonal
Effect of community-initiated kangaroo mother care on survival of infants with low birthweight: a randomised controlled trial	Mazumder *et al*	Home visits by intervention workers	NMR	Intervention workers	Mothers of LBW babies	KMC	Informational, relational
Effect of provision of home-based curative health services by public sector health-care providers on neonatal survival: a community-based cluster-randomised trial in rural Pakistan	Soofi *et al*	Home visits by CHWs	NMR	CHWs	Mothers of term babies	Community-based newborn care, including resuscitation, management of newborns, administering antibiotics and referrals.	Informational, Management, Interpersonal
Online participatory intervention to promote and support exclusive breastfeeding: randomized clinical trial	Cavalcanti *et al*	Facebook Group	Behaviour change	Research team	Mothers of term babies	BF	Informational, Relational, Interpersonal
Effect of home-based newborn care on neonatal and infant mortality: a cluster randomized trial in India.	Rasaily *et al*	Home visits by CHWs	NMR	CHWs	Mothers of term or LBW babies	Health education, management of newborns, treatment for minor illnesses and referrals.	Informational, Relational, Interpersonal
Training and evaluation of Community Health Workers (CHWs): towards improving maternal and newborn survival in an urban setting in KwaZulu-Natal, South Africa.	Ndaba *et al*	Home visits by CHWs	Behaviour change	CHWs	Pregnant women	Assess and support the mother during postdischarge, including newborn management and encouraging follow-ups.	Informational, Relational
Strengthening the Community Support Group to improve maternal and neonatal health seeking behaviors: a cluster-randomized controlled trial in Satkhira District, Bangladesh.	Gai Tobe *et al*	Group-based activities	Behaviour change	Community Support Groups (representatives from across the community).	Women who had given birth in the last year	Community mobilisation activities, planning for care (ANC to postnatal), referrals, education among residents.	Informational, Interpersonal
Newborn care practices at home: effect of a hospital-based intervention in Sri Lanka.	Senarath *et al*	Training hospital HCWs	Behaviour change	Hospital HCWs	Mothers of term babies	Education on newborn care	Informational
Adaptation of kangaroo mother care for community-based application	Quasem *et al*	Home visits by CHWs	Behaviour change	CHWs	Expectant or recently delivered women	KMC	Informational, Relational
Can mothers recognize neonatal illness correctly? Comparison of maternal report and assessment by community health workers in rural Bangladesh	Choi *et al*	Home visits by CHWs	Behaviour change	CHWs	Pregnant women	Education on neonatal danger signs and CHW management of newborns including administering antibiotics or referrals to facilities.	Informational, Management
Introduction of newborn care within integrated community case management in Uganda	Kayemba *et al*	Home visits by CHWs	Process	CHWs	Pregnant and recently delivered women	Essential newborn care, encourage follow-ups at facilities, identify newborn danger signs, advise on healthy newborn practices.	Informational, management, relational, interpersonal
Can a community health worker and a trained traditional birth attendant work as a team to deliver child health interventions in rural Zambia?	Yeboah-Antwi *et al*	Pairing CHWs and TBAs	Process	CHWs and TBAs	CHWs and TBAs	Improved teamwork between CHWs and TBAs	Relational, Interpersonal
Effectiveness of the baby-friendly community initiative on exclusive breastfeeding in Kenya	Kimani-Murage *et al*	Home visits by CHWs and support groups	Behaviour change	CHWs	Pregnant women aged 15–49	BF	Informational
Community-based father education intervention on breastfeeding practice - Results of a quasi-experimental study	Bich *et al*	Home visits, monthly meetings and media campaigns	Behaviour change	Midwives and CHWs	Expectant fathers	Education on BF	Informational, interpersonal
EMBRACE intervention to improve the continuum of care in maternal and newborn health in Ghana: The RE-AIM framework-based evaluation.	Kikuchi *et al*	CoC cards, training HCWs on CoC, retaining women for 24 hours after delivery and home visits	Process	HCWs in hospitals	Recently delivered women	Increasing completion of CoC	Management, relational
Scaling up Kangaroo Mother Care in Ethiopia and India: a multi-site implementation research study	Mony *et al*	Maximising access to KMC facilities, training and supporting facility staff, supporting KMC at home postdischarge	Behaviour change	HCWs in hospitals and CHWs in the community	Mothers of LBW babies	KMC	Informational, relational, interpersonal
Effectiveness of a Quality Improvement Program Using Difference-in-Difference Analysis for Home Based Newborn Care—Results of a Community Intervention Trial.	Dhanesh Goel *et al*	Home visits by CHWs and group based activities	Behaviour change	CHWs	Pregnant or recently delivered women	Health education on newborn care, including BF and danger signs	Informational, relational, interpersonal
mHealth intervention ‘ImTeCHO’ to improve delivery of maternal, neonatal, and child care services—A cluster-randomized trial in tribal areas of Gujarat, India	Modi *et al*	mHealth service	Behaviour change	CHWs and HCWs	Mothers of children under the age of 2	Videos with health messages for family members, screening checklist and referrals. HCWs can track high risk cases.	Informational, relational
LATCH Score at Discharge: A Predictor of Weight Gain and Exclusive Breastfeeding at 6 Weeks in Term Healthy Babies	Shah *et al*	Recording LATCH scores at discharge and giving extra support to mothers who need it	Behaviour change	Lactation nurse	Mothers of term babies	BF	Informational
Telemonitoring of high-risk neonates discharged from SNCU using a novel device: a pilot study	Madireddy and Lingaldinna	mHealth service	Process	Nurses	Parents of high risk neonates	Monitoring high risk babies and referrals	Management
Community health promotion and medical provision for neonatal health-CHAMPION cluster randomised trial in Nagarkurnool district, Telangana (formerly Andhra Pradesh), India	Boone *et al*	Home visits by CHWs	NMR	CHWs	Women in the community	Education on newborn care and increasing awareness of available services. Tracking high risk mothers and referrals.	Informational
Potential effectiveness of Community Health Strategy to promote exclusive breastfeeding in urban poor settings in Nairobi, Kenya: a quasi-experimental study	Kimani-Murage *et al*	Home visits by CHWs	Behaviour change	CHWs	Pregnant women^12–49^	BF	Informational, relational
Effect of the Uganda Newborn Study on care-seeking and care practices: a cluster-randomised controlled trial	Waiswa *et al*	Home visits by CHWs	Behaviour change	CHWs	Pregnant women	Education on newborn care including BF, skin-to-skin care, delayed bathing and care-seeking for illness.	Informational
Effect of implementation of Integrated Management of Neonatal and Childhood Illness (IMNCI) programme on neonatal and infant mortality: Cluster randomised controlled trial	Bhandari et al	Home visits by CHWs	NMR and behaviour change	CHWs	Recently delivered women	Education on newborn care practices, including breast feeding, thermal care, cord care, care-seeking for illness. Newborns were assessed for signs of illness and referred.	Informational, Management
Household surveillance of severe neonatal illness by community health workers in Mirzapur, Bangladesh: coverage and compliance with referral	Darmstadt *et al*	Home visits by CHWs	Process	CHWs	Pregnant women	Identifying severely ill newborns and referring them to care	Informational, management
Increasing access to care for sick newborns: evidence from the Ghana Newhints cluster-randomised controlled trial	Manu *et al*	Home visits by CHWs	Behaviour change	CHWs	Recently delivered women	Promoting early newborn care practices and providing referrals for sick babies	Informational, relational
Effect of women’s groups and volunteer peer counselling on rates of mortality, morbidity, and health behaviours in mothers and children in rural Malawi (MaiMwana): a factorial, cluster-randomised controlled trial	Lewycka *et al*	Group-based activities and home visits	NMR and behaviour change	Facilitators and peer counsellors	Pregnant women	Identify local problems and solutions. Promoting early newborn care practices including care-seeking.	Informational, interpersonal
Understanding how women’s groups improve maternal and newborn health in Makwanpur, Nepal: a qualitative study	Morrison *et al*	Group-based activities	Process	Local female facilitators	Women in the community	Identify local problems and solutions	Informational, Interpersonal
A community based approach to improve health care seeking for newborn danger signs in rural wardha, India	Dongre *et al*	Group-based activities	Behaviour change	Community-based organisations	Pregnant women	Community mobilisation and health education on care-seeking	Informational, Interpersonal
Community-based antenatal education in Istanbul, Turkey: Effects on health behaviours	Mulzan Turan and Say	Group education sessions	Behaviour change	Nurse, facilitator and trained community member	Pregnant women	Education on newborn care practices	Informational
Feasibility assessment of an ergonomic baby wrap for kangaroo mother care: A mixed methods study from Nepal	Thapa *et al*	Ergonomic KMC wrap	Behaviour change	HCWs at the hospital	Family of LBW babies	KMC	Informational, management
Linking Home Based New Born Care to the Existing Government Health system in Tamil Nadu: Pilot Study	Jeganathan *et al*	Integrated care	Process	HCWs and CHWs	Recently delivered women	Linking CHWs to facilities for referrals. Extra nurses, equipment (including ambulances) were provided at the hospitals.	Informational, Relational, Interpersonal
Effect of community-based behaviour change management on neonatal mortality in Shivgarh, Uttar Pradesh, India: a cluster-randomised controlled trial	Kumar *et al*	Home visits by CHWs	NMR and behaviour change	CHWs	Community stakeholders and pregnant women and their families	Essential newborn care, including BF, thermal care, cord care, care-seeking from professionals.	Informational, relational
Key lessons from a mixed-method evaluation of a postnatal home visit programme in the humanitarian setting of Gaza	de Vries *et al*	Home visits by nurses and midwives	Process	Midwives and nurses	First time mothers and mothers of high-risk babies	Improving continuity of care post-discharge	Relational
Explaining the impact of a women’s group led community mobilisation intervention on maternal and newborn health outcomes: the Ekjut trial process evaluation	Rath *et al*	Group-based activities	Process	Local facilitators	Women in the community	Identify local problems and solutions	Interpersonal
Effect of Village Health Team home visits and mobile phone consultations on maternal and newborn care practices in Masindi and Kiryandongo, Uganda: a community-intervention trial	Ayiasi *et al*	mHealth service Home visits by CHWs	Behaviour change	CHWs	Pregnant women	Education on essential newborn care	Informational, relational
Men’s knowledge and awareness of maternal, neonatal and child health care in rural Bangladesh: a comparative cross sectional study.	Nasreen *et al*	Group activities	Behaviour change	Programme organisers	Married men	Education on essential newborn care and referrals	Informational, interpersonal
Effectiveness of an integrated approach to reduce perinatal mortality: recent experiences from Matlab, Bangladesh.	Rahman *et al*	Home visits by CHWs	NMR	CHWs	Pregnant women	Improving links between communities and facilities via CHW counselling and referrals.	Informational, management
Assessing community based improved maternal neonatal child survival (IMNCS) program in rural Bangladesh	Rahman *et al*	Home visits by CHWs	NMR	CHWs	Pregnant women	Improving the continuum of care via CHW counselling and improving services at facilities.	Informational, relational
Testing a scalable community-based approach to improve maternal and neonatal health in rural Nepal	Hodgins *et al*	Home visits by CHWs	Behaviour change	CHWs	Mothers and family members	Education on essential newborn care	Informational, relational
Effect of scaling up women’s groups on birth outcomes in three rural districts in Bangladesh: a cluster-randomised controlled trial	Azad *et al*	Group-based activities and health system strengthening	NMR and behaviour change	Local facilitators	Recently delivered women^15–49^	Identifying local problems and solutions. Refresher training for HCWs	Informational, relational, interpersonal
Effect of community-based promotion of exclusive breastfeeding on diarrhoeal illness and growth: a cluster randomised controlled trial	Bhandari et al	Group education	Behaviour change	TBAs, CHWs and other health workers	Recently delivered women	Education on BF	Informational, interpersonal
Comparison of the effect of two systems for the promotion of exclusive breastfeeding	Coutinho *et al*	Baby friendly hospital initiative and home visits	Behaviour change	HCWs and CHWs	Recently delivered women	BF	Informational, relational, interpersonal
Exclusive breastfeeding promotion by peer counsellors in sub-Saharan Africa (PROMISE-EBF): a cluster-randomised trial	Tylleskar *et al*	Home visits	Behaviour change	Peer counsellors	Pregnant women	BF	Informational, relational
Can early postpartum home visits by trained community health workers improve breastfeeding of newborns?	Mannan *et al*	Home visits by CHWs	Behaviour change	CHWs	Pregnant women	Essential newborn care with a focus on BF	Informational, relational
Community-Based Kangaroo Mother Care to Prevent Neonatal and Infant Mortality: A Randomized, Controlled Cluster Trial	Sloan *et al*	Home visits by CHWs	NMR	CHWs	Pregnant women and their families	KMC	Informational
Postnatal peer counselling on exclusive breastfeeding of low-birthweight infants: A randomized, controlled trial	Agrasada *et al*	Home visits by CHWs	Behaviour change	CHWs	Mothers of LBW babies	BF counselling	Informational, relational
Impact of an integrated nutrition and health programme on neonatal mortality in rural northern India	Baqui *et al*	Home visits by CHWs	NMR	CHWs	Mothers who had given birth in the last 2 years	Information to encourage behaviour change for essential newborn care	Informational, relational
Education for expectant fathers in workplaces in Turkey	Sahip *et al*	Group education sessions	Behaviour change	Doctors attached to companies	Men working in large offices	Education on antenatal care, supporting partners, BF, postnatal check ups	Informational
The Effect of Increased Coverage of Participatory Women’s Groups on Neonatal Mortality in Bangladesh A Cluster Randomized Trial	Fottrell *et al*	Group-based activities	NMR	Local facilitators	Women in the community	Identifying local problems and solutions	Interpersonal
Effect of Postnatal Home Visits on Maternal/Infant Outcomes in Syria: A Randomized Controlled Trial	Bashour *et al*	Home visits by midwives	Behaviour change	Midwives	Recently delivered women	Education on newborn care, including BF, uptake of newborn care and immunisation	Informational, relational
Improvement of perinatal and newborn care in rural Pakistan through community-based strategies: a cluster-randomised effectiveness trial	Bhutta *et al*	Home visits by CHWs	NMR	CHWs	Women in the community. Pregnant women	Education on newborn care, including identification of danger signs and promotion of care-seeking	Informational, relational
Practice of skin-to-skin contact, exclusive breastfeeding and other newborn care interventions in Ethiopia following promotion by facility and community health workers: results from a prospective outcome evaluation	Callaghan-Koru *et al*	Counselling and home visits	Behaviour change	HCWs, CHWs and HEWs	Recently delivered women	KMC and BF	Interpersonal
Use of Job Aids to Improve Facility-Based Postnatal Counseling and Care in Rural Benin	Jennings *et al*	Job aids for postnatal care	Behaviour change	Nurse-midwives	Recently delivered women	Education on immediate newborn care, including danger signs	Informational, management
Effect on Neonatal Mortality of Newborn Infection Management at Health Posts When Referral Is Not Possible: A Cluster-Randomized Trial in Rural Ethiopia	Degefie Hailegebriel *et al*	Home visits by CHWs and health system strengthening	NMR	CHWs and HEWs	Pregnant women	Essential newborn care, including referrals. Health centres were given job aids, antibiotics and HCWs received training.	Management
Effect of an integrated community-based package for maternal and newborn care on feeding patterns during the first 12 weeks of life: a cluster-randomized trial in a South African township	Ijumba *et al*	Home visits by CHWs	Behaviour change	CHWs	Pregnant women (over 17)	BF	Informational, relational
Can a community health worker administered postnatal checklist increase health-seeking behaviors and knowledge?: evidence from a randomized trial with a private maternity facility in Kiambu County, Kenya	McConnell *et al*	Home visits and phone calls by CHWs using a checklist	Behaviour change	CHWs	Recently delivered women	Education on danger signs and referrals	Informational, management, relational
Assessment of the uptake of neonatal and young infant referrals by community health workers to public health facilities in an urban informal settlement, KwaZulu-Natal, South Africa	Nsibande *et al*	Home visits by CHWs	Behaviour change	CHWs	Pregnant women	Education on danger signs and referrals	Informational, management, relational
Effectiveness of conditional cash transfers (Afya credits incentive) to retain women in the continuum of care during pregnancy, birth and the postnatal period in Kenya: a cluster randomised trial	Vanhuyse *et al*	Cash transfer	Behaviour change	Nurses	Pregnant women	Increased care attendance	Relational

HCW, healthcare worker; KMC, kangaroo mother care; LBW, low birth weight; NMR, neonatal mortality rate; TBA, traditional birth attendant.

We prioritised a set of 28 core articles (which provided rich descriptions of intervention, results, contexts and outcomes) and used these as a starting point for iterative analysis of the 65 articles. Guided by the review questions, a more detailed analysis of the articles was underpinned by the continuity of care framework by Haggerty *et al* 29 which distinguishes between informational, management and relational continuity. Informational continuity is defined as information being shared and used appropriately; management continuity as managing an illness in a consistent and coherent manner; and relational continuity as the ongoing relationship between a patient and one or more providers. We treated these different facets of continuity as distinct logics, that is, assumptions underpinning how continuity might be achieved. In reviewing these papers, the lead author sought to interpret how different logics of continuity underpinned and informed different interventions in each of the studies. Multiple continuity logics could underpin a single intervention, for example, informational continuity might be attempted as part of an intervention while explicitly or implicitly supporting relational continuity. The logics underpinning continuity were not always clearly articulated by study authors, therefore, interpretation and judgement was needed. The first author conducted an inductive analysis of extracted data to compare and contrast findings across studies, interventions and continuity logics and produce summaries, which were then reviewed and discussed with coauthors. The analysis of core articles resulted in the addition of ‘interpersonal continuity’ as a new category, highlighting the importance of relationships between family members, between healthcare workers or between people within the community.

To address the third research question, the methods used in papers were assessed to see if they were participatory. Participatory studies are defined as involving the community^30 31^ and designing interventions with people, not for them.^32^ However, there are different levels of participation, for example, who is included (community members vs end-users vs stakeholders) and when they are involved (problem definition through to assessment of the project).^33^ We applied the classification by Vaughn and Jacquez^20^ to differentiate the participatory studies. The authors defined ‘empowerment’ of participants as the community leading research decision-making, while ‘informing’ participants was defined as information being provided to the community and ‘involvement’ of participants as researchers working directly with the community.

To address the fourth research question, we defined opportunities as structural or behavioural levers to continuity of care in the postdischarge period, and challenges as structural or behavioural challenges that are barriers to continuity of care in the postdischarge period. GG extracted information on opportunities and challenges from the studies that were considered pertinent to the implementation of the intervention, then conducted an inductive thematic analysis.

## Results

### Description of the studies

**Figure 1:**
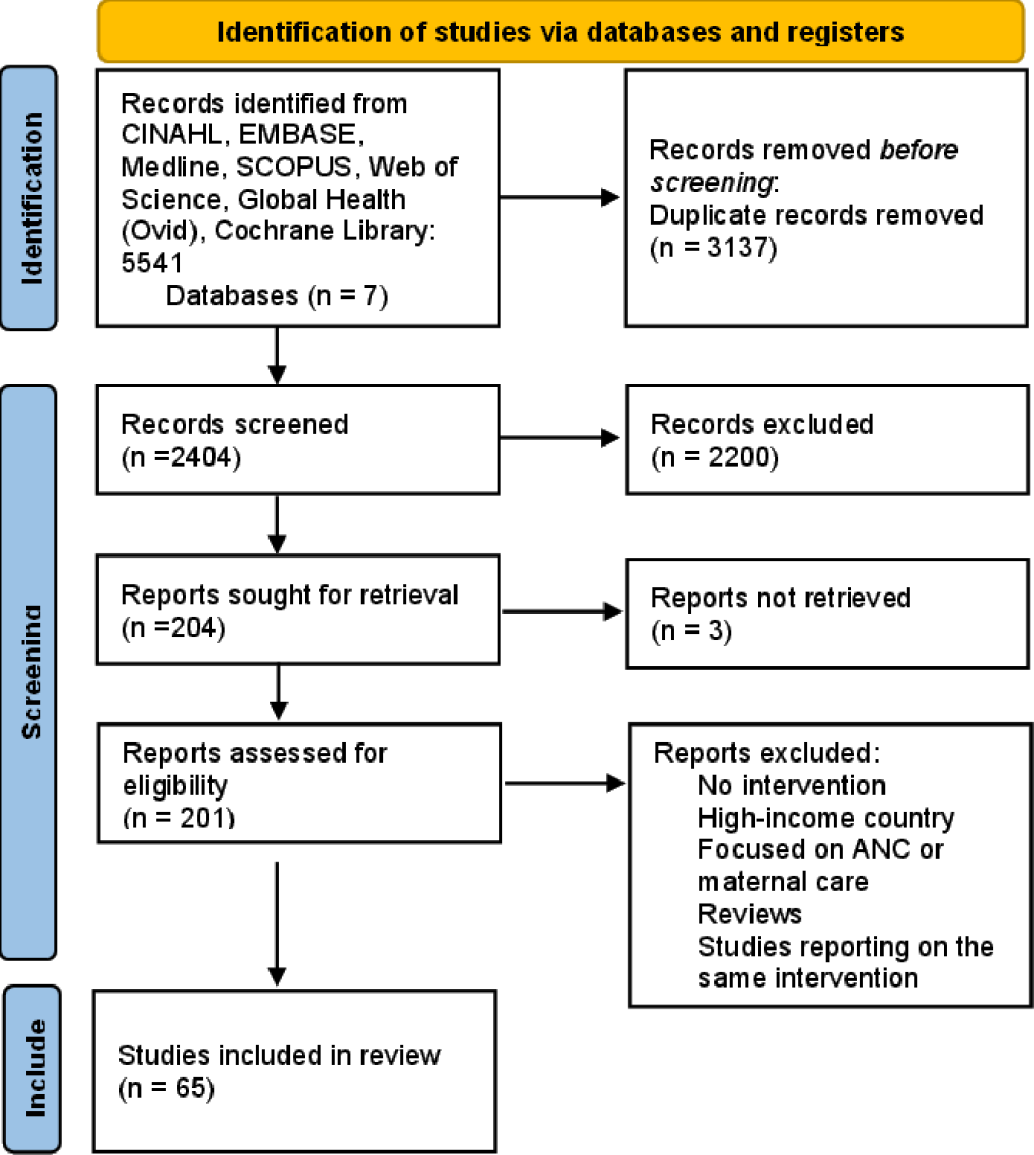
PRISMA chart demonstrating the sources of data selected for review. ANC, antenatal care; PRISMA, Preferred Reporting Items for Systematic Reviews and Meta-Analyses.

The database search produced 5541 records, of which 3137 were removed as duplicates and 2404 were screened by title and abstract. 204 articles were included in full-text review, resulting in 65 articles in the final dataset. A Preferred Reporting Items for Systematic Reviews and Meta-Analyses extension for Scoping Reviews diagram in figure 1 summarises the search results and screening processes for this study.

### Study characteristics

**Figure 2:**
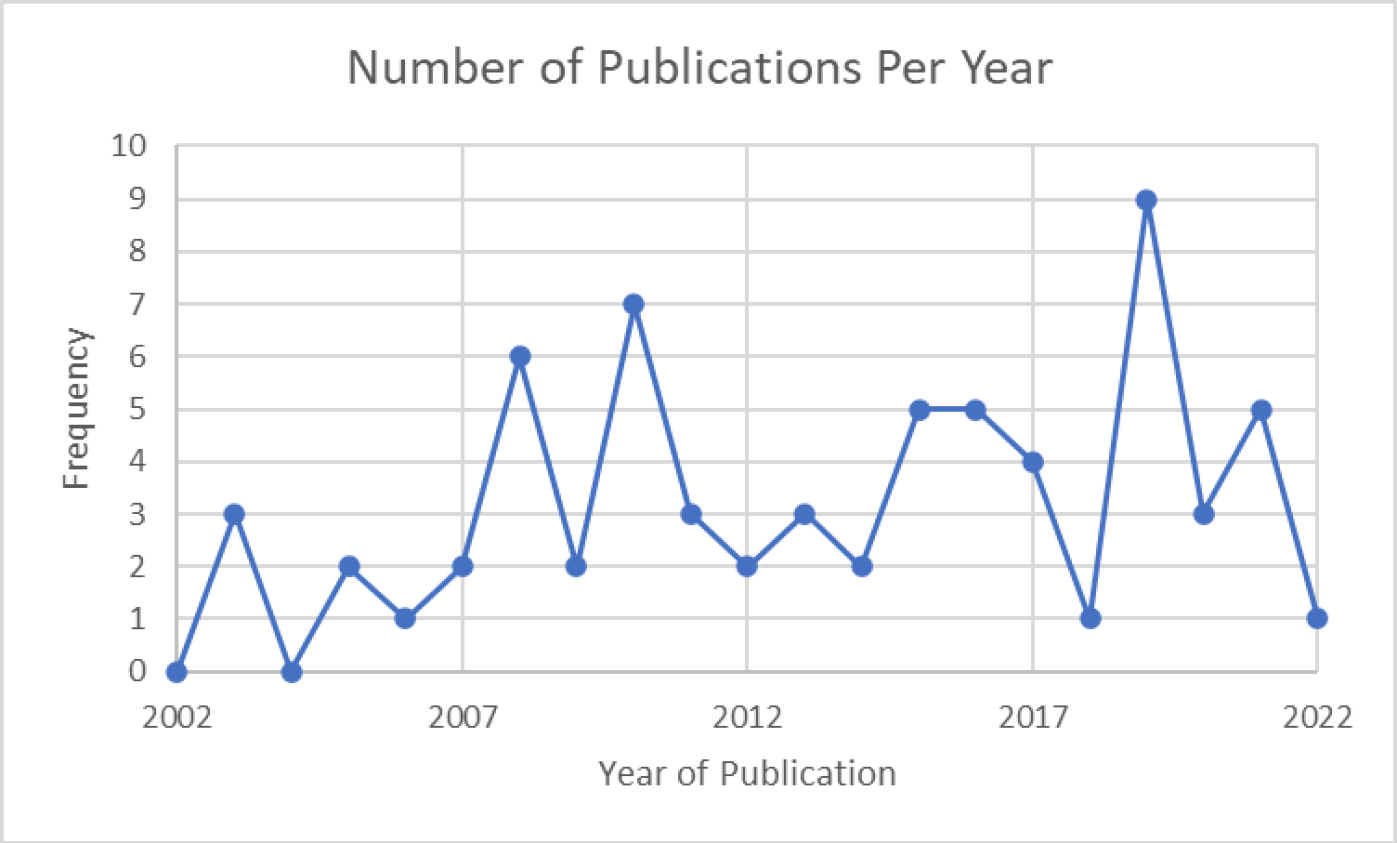
The number of articles published per year on continuity of care for newborns in resource-constrained settings, that is, LMICs. LMICs, low-income and middle-income countries.

**Figure 3:**
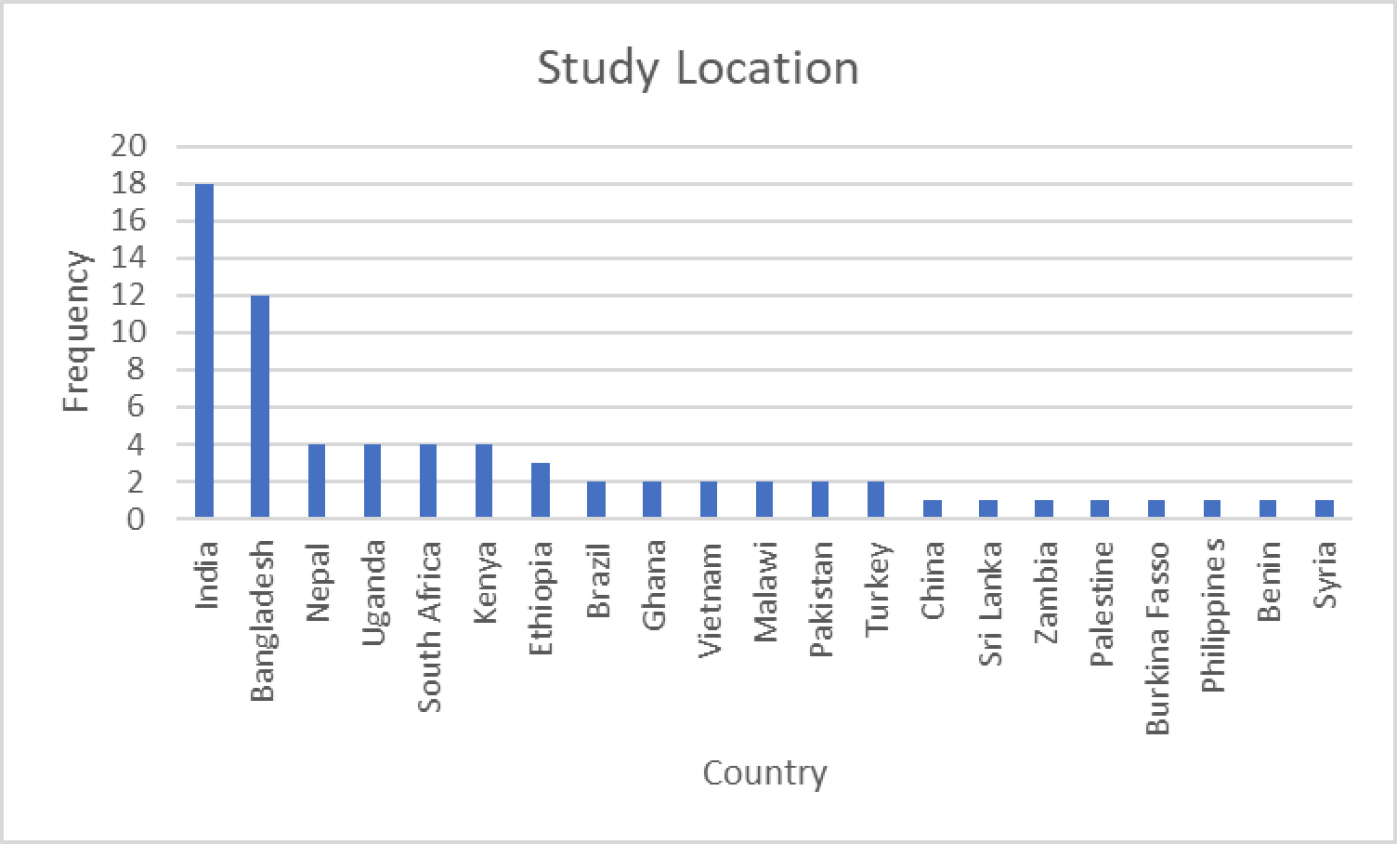
Countries where studies on continuity of newborn care were conducted.

All articles were published from 2003 onwards, with fluctuations in the number of articles per year, as shown in figure 2. Figure 3 shows the countries where the studies were conducted. Just under two-thirds of the articles were conducted in Asia and just under a third in Africa, with two articles each from South America and the Middle East.

The final dataset included 44 quantitative articles (68.2%) and 7 mixed-methods articles (10.6%). The 14 remaining articles (21.2%) were participatory and described how the community was engaged. Of the participatory studies, only one study was qualitative, using interviews, observations and focus-group discussions. Two studies had qualitative components, including interviews or observations and focus group discussions (FGDs). Quantitative studies mostly consisted of cluster randomised controlled trials (RCTs) (27/65; 41.5%) or RCTs (14/65; 21.5%). The seven mixed-methods studies used surveys combined with in-depth interviews or focus group discussions.

### Interventions

As table 1 shows, more than half (56.9%) of the 65 interventions in the articles reviewed involved home visits from community health workers (CHWs), and a small number (4.6%) involved home visits from midwives.^34–36^ About 20% of all studies (18.5%) focused on newborn care education during home visits, such as informing mothers about danger signs, care-seeking and thermal care.^36–47^ A further 20% (18.5%) of studies focused on providing community-based newborn care during home visits, which could include administration of antibiotics or resuscitation, management of newborns or referrals to care.^37 48–58^ Nine interventions (13.8%) involved group-based activities such as plan-do-study-act (PDSA) or participatory action cycles^59–67^ Seven studies (10.8%) included healthcare worker training as an intervention.^68–74^

Most of the 65 interventions (61.5%) involved CHWs conducting home visits as mentioned earlier. Other healthcare workers such as doctors, nurses and midwives, were involved in 17 studies (26.1%),^9 35 36 68–72 74–81^ and 8 studies (12.3%) involved facilitators who were locally recruited and trained.^59 60 62 63 65–67 80^

The majority of studies (36.9%) targeted pregnant women,^59 60 62 63 65–67 80^ followed by mothers of term babies (35.3%).^37 42 44 46 53 54 58 60 64 80–90^ Only nine studies (13.8%) targeted mothers of babies who were high risk, that is, low birth weight (LBW) or preterm.^35 48 50 51 73 74 77 91 92^ 36 studies (55.4%) aimed to modify behaviours that contribute towards reducing neonatal mortality, such as breastfeeding and kangaroo mother care (KMC).^34 36–38 40 42–45 47 53 56 61 64 68–74 76 78–84 86 89 91 93–96^ 15 studies (23.1%) explicitly targeted neonatal mortality^39 46 48–52 58 60 67 87 88 90 92 97^ and 4 studies (6.2%) targeted both neonatal mortality and behaviours that contribute towards reducing neonatal mortality.^41 57 62 66^ Ten studies (15.4%) focused on the implementation process itself.^35 54 55 59 63 65 75 77 98 99^

### Participatory studies

Of all studies, 16.9% (11/65) were defined by study authors as participatory (see table 2), and 3.1% (2/65) were not defined as participatory, but used participatory elements in intervention design to different degrees, for example, consulting with key stakeholders, such as district health management, before implementing KMC interventions and essential newborn care.^40^ Participatory learning and action cycles were used most to facilitate participation (6/11; 54.5%),^61–63 65–67^ with only two studies (2/11; 18.2%) using PDSA cycles.^59 60^ The participatory learning and action cycle activities led to interventions such as disseminating information via counselling women in their homes or in community centres,^59^ bicycle ambulances^60^ and health education on postnatal care.^59 60^ A small number of studies (2/11; 18.2%) focused on community mobilisation.^61 64^

**Table 2:** General characteristics of participatory studies included in the review

Author	Self-defined as participatory?	Method	Who was involved?	Level of involvement	What was done?	What was the outcome?	How was the outcome measured?
Eriksson *et al*	Yes	Plan-do-study-act	Facilitators were recruited from the Women’s Union and had to be mothers. Each group included: the vice chairperson of the people’s committee (responsible for education and health in the commune), three members of staff from the community health centre, one CHW and a Women’s Union representative from the commune or village and a population collaborator (responsible for collecting population data and performing family planning.	Empower	Maternal and neonatal health groups were created, facilitators helped in monthly meetings. Members were meant to be supported and empowered to identify local problems and actions related to neonatal health.	In both groups with facilitators (low and high ratings) the NMR was lower than control, but the high group had a significantly lower or of neonatal mortality.	Process data was taken from notes by facilitators, at meetings and supervisor’s records of meetings with facilitators.
Colbourn *et al*	Yes	Plan-do-study-act	Pregnant women from the community, volunteer facilitators, maternal and neonatal health task force (unlcear who was involved in this).	Empower	Participatory women’s groups were created to mobilise communities around maternal and newborn health. The groups followed an action cycle to identify and prioritise maternal and neonatal health problems, decide on local solutions, advocate for, implement and evaluate such strategies (Facility improvement was also done. The study followed a 2×2 factorial cluster RCT.	NMR was 22% lower in facility and community intervention than control clusters and the perinatal mortality rate was 16% lower in community intervention clusters	Village volunteers collected data on pregnancies, miscarriages, abortions, stillbirths, live births, neonatal deaths, maternal deaths and place of delivery using standardised forms.
Cavalcanti *et al*	Yes	Participatory intervention using an online social network	The research team (academics in nutrition, nursing, social work and psychology; nutritionists; paediatricians; professors). Brazilian MoH and WHO recommendations were used.	Inform	The research group created a booklet on BF with the MoH and WHO. The intervention was a Facebook group where topics from the booklet were posted each week and discussion was encouraged. The research team responded to any questions.	EBF rates were higher throughout the study compared with the control group. The median number of days of EBF was higher in the intervention group than in the control.	Surveys via phone calls with mothers (self-reported).
Gai-Tobe *et al*	Yes	Participatory learning action cycle	Facilitators (government CHWs), community support groups (housewives, persons with disabilities, elderlies, adolescents, religious leaders and freedom fighters), the wider community (educated by CSG), pregnant women.	Involve	Community support groups were set up to implement activities to support safe motherhood, including promoting birth planning, ANC, PNC and neonatal care counselling and referrals. Outside of the group community diagnosis and resource mapping exercise and advocacy and planning meetings were held at union level.	Home deliveries decreased in the intervention group. ANC, PNC provided after 42 days and care for sick newborns from skilled professionals increased	Baseline and endline surveys.
Mony *et al*	No	Formative research was used to inform an intervention to scale up use of KMC	State and district government health managers and a local research institution.	Consult	Discussions were held with government officials about policies, experiences and required resources.	Coverage was 25% after model 1, 40% after model 2, close to 55% with model 3. KMC was initiated for 82% eligible infants overall, 52% continued to receive KMC after discharge.	Qualitative data on feedback from programme learning (managers, health providers and women/family responses) and quantitative data on KMC coverage.
Waiswa *et al*	No	Design workshop and formative research	National policy-makers, experts and the district health management teams.	Consult	A design workshop was held with national policy-makers and experts and the district health management teams of the trial districts. The intervention was then piloted.	Significant improvements in birth preparedness and essential newborn care practices, including breast feeding, hygienic cord care and thermal protection practices associated with reduced neonatal mortality.	Baseline and endline surveys.
Lewycka *et al*	Yes	Participatory action cycle	Local facilitator, pregnant women, men from the community.	Empower	The facilitators used a manual to implement the cycle with participatory rural appraisal methods and picture cards of maternal and newborn health problems to guide discussion. Members identified and prioritised maternal and child health problems, identified strategies to implement, planned and implemented them, and assessed them and made plans for the future.	Women’s group interventions reduced MMR, PMR and NMR in years 2 and 3. EBF also increased.	Questionnaires used during home visits.
Morrison *et al*	Yes	Participatory learning action cycle	Local facilitator, women in the community.	Empower	Women’s groups were created to discuss and exchange ideas around maternal and newborn health.	30% reduction in neonatal mortality in intervention areas. The groups learnt, developed confidence, disseminated information within communities and increased community capacity to take action. Women’s groups were a source of support, people could share ideas and experiences and learn. Participants become confident, expressed themselves openly and without shame. Some evidence of standing up to norms, for example, intercaste marriage. Women spread information in the community to non-group members, which improved hygiene and immunisation.	FGDs (including photoelicitation) with women in the groups, women not in the groups, mother-in-law, men and CHWs. Observations of group sessions. Interviews with facilitators, CHWs and community leaders.
Dongre *et al*	Yes	Participatory community mobilisation	Women, adolescents, farmers, local female CHW, local self-government.	Inform	Women’s self-help groups, adolescent girls groups, farmers groups and village coordination committees (VCCs) (made up of community groups) to form a platform at community level for action. Capacity of the VCC members was strengthened during their monthly village-based meetings to develop their gender sensitive village health plan with culturally appropriate solutions to local problems and develop emergency transport plan. The trained social worker facilitated Community’s Action Experience Learning Cycle through VCCs to explore and collectively act on their priority maternal and child health issues.	Women’s membership in self-help group and health insurance coverage of family significantly improved from 8% to 16.9% and 23.3% to 58.6%, respectively. There was significant improvement in mothers’ knowledge regarding newborn danger signs.	Lot Quality Assurance Sampling method - quant surveys.
Kumar *et al*	Yes	Codevelopment of a community-based intervention for behaviour change management	Village heads, community leaders, respected members, priests, teachers, traditional newborncare providers and birth attendants, unqualified medical practitioners and health system workers, pregnant woman or mother, mother-in-law, father-in-law and husband.	Involve	A community-based intervention for behaviour change management was designed. Researched aimed to understand existing practices, design relevant behaviour change messages, create a shift in reasoning in favour of improved practices, negotiate barriers to change by optimising available resources and providing viable alternatives, equip households with necessary skills, build self-confi dence and create a supportive environment.	NMR reduced in both intervention groups by over 50%. Significant improvements were seen with targeted newborn-care practices. Commencement of early BF was higher in the intervention groups than in the control. Care-seeking from unqualified medical practitioners decreased in the intervention group.	Baseline and endline surveys.
Rath *et al*	Yes	Participatory learning and action cycle	Women from the community, local facilitators.	Empower	Groups met monthly to discuss issues around pregnancy, childbirth and the postnatal period. The cycle emphasised collective problem solving and planning and was divided into four phases. Group members organised community meetings at specific times during the cycle to share their learning with the wider community and enlist their support in implementing strategies to address problems in pregnancy and childbirth.	Reduction in NMR. The interventions were locally accepted, used participatory approaches to develop knowledge, skills and ‘critical consciousness’, community involvement beyond the groups occured. The intervention was successful because facilitators were local, locally appropriate discussion materials were used, and timing and content of meetings were flexible.	Case studies, direct observation of meetings and FGDs, quantitative data such as women’s group meeting attendance records and data from the trial’s main monitoring and evaluation questionnaire for information on group membership status.
Azad *et al*	Yes	Participatory learning and action cycle	Local facilitator, women who has recently given birth.	Empower	The facilitator activated and strengthened groups, to support them in identifying and prioritising maternal and neonatal problems, to help to identify possible strategies and to support the planning, implementation and monitoring of strategies in the community.	Participatory women’s groups did not significantly reduce neonatal mortality	Prospective monitoring system with TBAs.
Fottrell *et al*	Yes	Participatory learning and action cycle	Women whose childbirth or death was recorded, men, TBAs, health service providers, teachers and community leaders.	Empower	New groups were formed and old groups (previous study) were involved. New groups had meetings on maternal and newborn health, old groups expanded into health for children under 5.	NMR was lower in the intervention group than clusters	A prospective surveillance system recorded all deaths during pregnancy and for up to 6 weeks postpartum and all births taking place in the study areas and their outcomes.

ANC, antenatal care; CHW, community health worker; EBF, exclusive breast feeding; KMC, kangaroo mother care; MoH, Ministry of Health; NMR, neonatal mortality rate; PNC, postnatal care; RCT, randomised controlled trial; TBA, traditional birth attendant.

Stakeholder participation varied: 6 of the 11 studies defined by authors as participatory (54.5%) included women from villages where the intervention was taking place, that is, pregnant women, recently delivered women or women of reproductive age.^60 62–67^ A few studies (3/11; 27.2%) involved representatives from the community such as CHWs, teachers and community leaders without always including women from the community.^57 59 61^ Using the classification by Vaughn and Jacquez,^20^ we found that seven studies aimed to ‘empower’ participants by engaging with them to define local problems, coming up with solutions, implementing them and evaluating whether they were effective.^59 60 62 63 65–67^ Two studies aimed to ‘inform’ participants via online social networks about breastfeeding^93^ and community health education about danger signs via women’s groups, farmers groups and village coordination committees.^64^ Two studies aimed to ‘involve’ participants, one by designing a behaviour change intervention via input from participants such as current newborn care practices, designing behaviour change messaging, optimising available resources and providing viable alternatives.^57^ Another study set up community support groups to implement activities supporting postnatal care, neonatal care counselling and referrals.

### Continuity logics

Each of the interventions was underpinned by specific assumptions (implicitly or explicitly) in terms of how to establish the conditions necessary for improving continuity of care for newborns in the community. Some interventions were underpinned by multiple, overlapping continuity logics. Looking at the most dominant logic from each paper, most interventions (53/65; 81.5%) targeted informational continuity, followed by relational continuity (33/65; 50.8%), our new category of interpersonal continuity (24/65; 36.3%) and management continuity (15/65; 23.1%). Results are presented under each of these categories below. Studies with more detail were included as an example from each continuity logic to illustrate the application and outcomes of the logic.

### Informational continuity

Informational continuity refers to information being shared and used appropriately, which means considering patient preferences, values and context, as well as medical records.^29^ This approach to organising care was the most dominant, used in 81.5% of studies (53/65). Informational continuity was evident in interventions that focused on sharing information via education sessions between CHWs, who had received training, and the community (23/53; 43.4%). The interventions attempted to improve neonatal outcomes by increasing knowledge on essential newborn care,^44 48 51 57 63 64 66 95^ danger signs,^49 54^ exclusive breast feeding (EBF), KMC,^74 79 86 92^ the importance of postnatal visits^78^ and providing referrals to care,^52 55 56 87^ which could include referral cards which mothers were encouraged to take to each visit.^55 56^

#### Opportunities

These interventions intended to improve the way information was shared, and studies reported they were more accepted in regions where CHWs were well established. Publications reported information sharing was easier because the community trusted CHWs and the content of the health messages they delivered.^44 52 54 83^ Information sharing was also reportedly improved by these interventions in settings where seeking care at facilities, for example, for deliveries or postnatal care, was uncommon.^48 49 51 52 57 63 66 74 86^ Publications reported information sharing was easier because home visits provided information to families where previously they would have received nothing.

#### Challenges

Sharing information was less easy in circumstances with patriarchal gender norms because men or mothers-in-law were considered the custodians of women’s access to health.^63 64 86 95^ However, two studies focused their education sessions on men, with the aim of increasing their knowledge and improving their decision-making ability^95^ or improving healthy behaviours and communication between couples.^78^

For example, Colbourn *et al* 60 combined quality improvement at facilities with participatory women’s groups via a two-by-two factorial design to see if these influenced perinatal and neonatal mortality. Groups followed an action cycle to identify and prioritise maternal and neonatal health problems, pick local solutions, implement and evaluate them; groups were facilitated by volunteer facilitators. Half the groups chose to focus on maternal and neonatal health knowledge and thus had maternal and neonatal health taskforces added to the groups. The taskforce aimed to promote delivery at facilities, postnatal care and provide health education. Quality improvement at facilities consisted of small tests of change using PDSA cycles and training healthcare workers on specific clinical areas such as neonatal resuscitation drills. There was a 22% reduction in neonatal mortality in the combined intervention clusters compared with the control arm.

### Relational continuity

Relational continuity refers to the ongoing relationship between a patient and one or more providers, with the understanding that consistency of core staff can provide a sense of coherence.^29^ This approach to organising care was evident in interventions that focused on repeated interactions (13/33; 39.4%), that is, home visits by care providers^35 44 48 50 51 54 56 57 69 74 83 86 92^ because they attempted to build trust with families and improve access to care for communities who would not otherwise receive a postdischarge follow-up.

#### Opportunities

These interventions intended to improve the patient–provider relationship and it was reported they were easier to implement in communities where there was greater acceptance of CHWs as they were carefully selected from and resided within the community,^44 48^ for example, communities showing appreciation for CHWs by providing transport or money.^54^ Some studies reported that the patient–provider relationship was improved by home visits by CHWs, as these communities reported a common practice of confining mothers and babies at home for 4–6 weeks after delivery, which is the period when babies are most likely to experience issues.^57 92^

#### Challenges

However, it was difficult to improve the patient–provider relationship in communities that faced difficulties physically accessing facilities due to geographical barriers such as mountains,^50^ distance to the facility^56^ and weak transport links.^86 99^ Relational continuity was also disrupted by women returning to their parental home for delivery, which may be far from their antenatal care facility.^66^

For example, de Vries *et al* 35 report impacts of relational continuity in neonatal care. This study describes how the Ministry of Health and two civil society organisations in Palestine trained nurses and midwives on the national postnatal care protocol including home-based postnatal care, neonatal care, postpartum complications and behavioural change communication. Nurses and midwives were given kits for home visits and conducted the first visit within 48–72 hours postdischarge. Relational continuity was built as the next two visits could be conducted at home or in a clinic with the same healthcare provider. The number of home visits for registered births increased from 5.4% to 12.9%. NMR remained the same as preintervention but qualitative interviews with both mothers and healthcare workers revealed that they thought the home visits led to increased support and thus better outcomes, though not in terms of NMR. Qualitative data showed that women felt encouraged to breastfeed and learnt about the benefits of breast feeding; EBF rates increased by 8.6% after the introduction of the programme. Healthcare workers said the visits made them more empathetic, and the mothers said they felt valued and cared for.

### Interpersonal continuity

Interpersonal continuity refers to relationships between family members, between healthcare workers or relationships within the wider community. Building these relationships outside of the ‘patient–provider’ dyad can foster support systems and lead to improvements in care. This approach to organising care was evident in interventions that focused on healthcare workers working together (2/24; 8.3%) because they attempted to improve communication between different cadres of staff.^52 54^ Interpersonal continuity was seen in interventions that used CHWs to foster supportive relationships within families (7/24; 29.2%) to promote gender equity via shared household chores,^69^ supportive relationships from husbands^34 95^ and joint decision-making between couples. Interpersonal continuity was encouraged by interventions which involved community mobilisation (8/24; 33.3%), which aimed to improve discussions within the wider community on localised issues related to maternal and newborn health.^49 51 59 63–65 74 93^

#### Challenges

Although these interventions intended to improve interpersonal relationships, findings show that they were less able to do so in communities with patriarchal cultures. This was reportedly due to women’s low levels of education and perceived low social standing,^64^ women’s access to health being gatekept by men (and therefore often not prioritised)^95^ and sociocultural norms against women seeking care.^66^

#### Opportunities

More participatory than non-participatory studies reported improvements among interpersonal relationships within the community, though this was only evident in studies that involved communities, as opposed to a focus on key stakeholders only. Improving relationships within the community via participatory methods was easier as facilitators were selected from the community to guide contextual intervention creation, and members of the community who were part of the intervention would often help those who were not.^63 65^

For example, Bich *et al* 34 designed a year-long community‐based educational intervention targeting fathers at antenatal, delivery and postnatal periods for supporting breastfeeding practices up to 6 months in Vietnam. The intervention was integrated into routine healthcare services, messages were played on loudspeakers, and flyers, mugs and calendars were also used to provide guidance for fathers so they could better understand breast feeding and be motivated to encourage continuity of EBF after early initiation. CHWs were trained to run monthly group health education and counselling, individual counselling at birth and at four follow-up home visits and monthly social public activities. These activities were geared to sensitising fathers to breastfeeding and encouraging them to be supportive partners for early initiation and continuation of breastfeeding. A contest was held to see which father retained the most knowledge and was the most supportive; the event was open to fathers who participated in the intervention and their family to make paternal engagement in EBF normalised, thereby improving interpersonal continuity. Early initiation of breast feeding (1 hour after birth) was 48.6% in the intervention group and 35.7% in the control group, and EBF remained higher in the intervention than in the control group up to 6 months.

### Management continuity

Management continuity refers to managing an illness in a consistent and coherent manner, which is particularly important when there are multiple care providers and there is a need for complementary and timely provision of care.^29^ This approach to organising care was evident in interventions that focused on introducing algorithms for CHWs based on national or WHO guidelines on when to treat or refer sick newborns^49 55 87^ or introducing protocols on the management of illness for healthcare workers^52 54 99^ (6/15; 40.0%). These interventions attempted to standardise and make consistent the care that newborns would receive.

#### Opportunities

Although these interventions intended to improve the way illnesses were managed, publications reported they had high rates of acceptance in circumstances where relationships had been previously built with the community because CHWs had been well established in the community for over 20 years.^48^ An example of ready acceptance is the community appreciating the care received by CHWs, and expressed this by providing gifts, lifts or money.^54^ In addition, interventions were reported to be easier to implement when researchers had been collecting data and running programmes for years.^87^

For example, Darmstadt *et al* 55 trained CHWs on pregnancy surveillance, essential newborn care, neonatal illness surveillance and management of illness based on a clinical algorithm adapted from Integrated Management of Childhood Illness in order to examine effects on NMR and the factors associated with coverage of postnatal assessments and compliance with CHW referrals. CHWs identified pregnancies and were notified when the woman was in labour so they could either be present or visit early in the postnatal period. CHWs conducted postnatal visits where they completed a standardised newborn assessment form, to identify serious illnesses requiring referral and made referral to the hospital according to the algorithm. CHWs’ classification of neonates with illness had high validity compared with physicians’ classification. NMR was 55.3 per 1000 for babies who had never been assessed by a CHW and 12.3 per 1000 for those who had been assessed at least once.

## Discussion

This scoping review aimed to map and describe interventions used in LMICs to improve continuity of care for newborns by examining which interventions have been used; to what extent participatory methods were employed to develop these interventions; how interventions attempted to establish continuity (ie, through what logics and assumptions) and what opportunities and challenges they encountered. Our findings show that the literature has primarily focused on quantitative studies, and studies in Asia, whereas the burden of neonatal mortality is greatest in Africa.^5^ Home visits were the most common intervention, and usually focused on providing education for mothers or community-based care for newborns. Other reviews have also recognised the importance of community-based care.^100 101^ Most interventions targeted pregnant women; however, one study suggested the optimal time to begin the continuum of care is prepregnancy.^102^ Few studies targeted mothers of babies who were high risk, such as those born preterm, LBW or sick.

Our analysis used the framework by Vaughn and Jacquez^20^ to distinguish different types of participatory approaches. Studies enacted participation by families, healthcare workers and other stakeholders to different degrees. Seven studies used PDSA, participatory learning or action cycles to ‘empower’ communities to come up with solutions to local issues.^59 60 62 63 65–67^ These studies involved representatives and/or women from the community in participatory learning and action cycles or PDSA cycles, where groups identified problems, came up with solutions, implemented their ideas and assessed whether they worked. Previous research has shown that women’s groups are an effective way of reducing NMR.^101 103^ While these studies involved members of the community in participatory learning and PDSA cycles, a review focused on RCT studies in high-income countries found that none of them involved participants in coproduction of study design or intervention.^104^ In our review, studies did not always engage mothers,^59^ which is problematic when interventions are described as participatory yet do not include the primary carers of newborns. Furthermore, the process of engaging community members as opposed to key stakeholders in interventions can foster continuity of care by improving interpersonal continuity. The level of participant engagement varied across studies, and it was not always clearly described, with the lowest level of engagement focusing on how to ‘inform’ participants, for example, providing information to participants. While informational continuity was the most applied logic, the earlier example by Colbourn *et al* 60 highlights that participatory studies can be used to engage with participants to understand their information needs before implementing an intervention. Detailed information about participant engagement is required to understand its role in study design and coproduction of interventions. Across papers, detailed information for replicability is lacking; how specific interventions were developed by groups^66 67^ and participatory involvement processes were not well described.^57 64^ These findings echo the call for future research papers to focus on the process involved with participatory approaches, especially in LMICs.^21^

In our analysis, we used the continuity framework by Haggerty *et al* 29 which distinguishes between three types of continuity logics (informational, relational and managerial). The literature on postdischarge care emphasised informational continuity more by focusing on information flow between the health system and carers, that is, education of mothers and families via CHWs. Sharing information with mothers and families contributed to improvements in knowledge on breast feeding and danger sign recognition. However, informed carers are not all that is required for good outcomes, and access to quality, affordable care must remain a priority. The focus on information sharing aligns with a review of continuity of care in high-income countries, which suggested further research into when and how to share information with mothers, as they received inconsistent advice and were not confident in care provision.^17^ In addition, information sharing and communication between healthcare workers and mothers was highlighted as a major issue during antenatal visits in four developing countries.^105^ Although the Haggerty framework covers relational continuity in terms of patient–provider relationship, this does not extend to relationships between healthcare workers, family members and mothers and the wider community. These relationships may impact continuity of care in LMICs, where men and mothers-in-law often have the final say in care-seeking.^63 64 86 95^ Recently literature has focused on people outside the mother–child dyad, with growing recognition that including men is important.^106 107^ Involving men in postnatal care is recommended by the WHO^108^ and can help tackle stereotypical gender norms.^109^ Therefore, we have extended the continuity framework by adding interpersonal continuity as a new category to emphasise that newborn care involves multiple complex relationships which need to be supported. Although a previous framework on continuity of care also recognises the importance of co-ordination between healthcare workers,^15^ the importance of family members in decision-making and care-seeking has been less explored.

### Strengths and limitations

In conducting this review, we searched a large number of databases to identify a range of different types of interventions aimed at improving continuity in postdischarge care. Beyond mapping interventions and their characteristics, we used and extended an established theoretical framework on care continuity, to delve deeper in the analysis and interpret how interventions attempt to establish continuity, as well as providing a critical assessment of participatory methods used. One of the limitations of this review is that grey literature was not included which means we may have missed some interventions not described in academic publications. In addition, there were missing descriptions of approaches used, such as how participatory methods were employed. Another challenge was the diversity of study types and reported outcomes which precluded direct quantitative comparisons, but enabled us to perform rich interpretive analysis, which was the aim of the review.

### Implications for practice and research

This scoping review may be of interest to policymakers, researchers and funders, when considering how different continuity interventions may best meet specific context(s) and what assumptions need to underpin these interventions. For example, home visits were the most popular intervention in the literature, but best outcomes were achieved in contexts where CHWs were well established and trusted within the community (ie, using informational and interpersonal continuity logics). In addition, some interventions faced challenges with implementation in contexts with patriarchal gender norms, hence more effort may be required for community engagement or interventions should be designed to include male-identifying community members in these settings. Some studies suggested focusing on babies at higher risk post discharge to improve outcomes^10 110^ and to be cost-effective.^111^ Further research is required on how to develop interventions to specifically meet continuity needs of high-risk newborns. Qualitative research could provide in-depth insights on contextual acceptability, which would enhance the ability to translate interventions across contexts. We are currently conducting a review of grey literature on continuity interventions in LMICs to understand if there is additional learning to be generated from studies that have not been published in academic journals.

## Conclusion

Most reports in the academic literature that describe research on the continuity of postdischarge care for newborns in LMICs are about home visits by CHWs, who educate mothers on newborn care practices (ie, assuming that improved information flow will result in better care) or provide community-based care. Most studies were quantitative and focused on infants who were born at full-term and healthy. This study adds to Haggerty and Reid’s continuity of care framework by including aspects of interpersonal continuity that are important in settings where relationships between family members, between healthcare workers and within the wider community are likely to influence intervention success. The review highlights that future efforts to reduce neonatal mortality and morbidity should focus further on high-risk neonates after hospital discharge in LMIC settings, as they often have the worst outcomes.

10.1136/bmjgh-2023-012894.supp2 Supplementary data

## Data Availability

Data are available in a public, open access repository.
