# Supplementary material for: Scoping review of interventions to improve continuity of postdischarge care for newborns in LMICs
Source: BMJ Glob Health. 2024 Jan 10;9(1):e012894. doi: 10.1136/bmjgh-2023-012894 (PMC10806884; doi:10.1136/bmjgh-2023-012894)
Supplement: Supplementary data [file bmjgh-2023-012894supp002.pdf]

General	
**Title**	**Author**
Intermittent kangaroo mother care and the practice of breastfeeding late preterm infants: results from four hospitals in different provinces of China	Zhang et al.
Breast crawl at birth, effect on breastfeeding rate and infant growth in infants delivered at an urban tertiary care public hospital: A randomized controlled trial	Mulupuru et al.
Lower mortality is observed among low birth weight young infants who have received home-based care by female community health volunteers in rural Nepal	Neupane et al.
Effect of community-based newborn-care intervention package implemented through two service-delivery strategies in Sylhet district, Bangladesh: a cluster-randomised controlled trial	Baqui et al.

Process evaluation of a knowledge translation intervention using facilitation of local stakeholder groups to improve neonatal survival in the Quang Ninh province, Vietnam.	Eriksson et al.
Low birth weight and preterm neonates: can they be managed at home by mother and a trained village health worker?	Bang et al.
Effects of quality improvement in health facilities and community mobilization through women's groups on maternal, neonatal and perinatal mortality in three districts of Malawi: maiKhanda, a cluster randomized controlled effectiveness trial	Colbourn et al.

Effect of community-initiated kangaroo mother care on survival of infants with low birthweight: a randomised controlled trial	Mazumder et al.
Effect of provision of home-based curative health services by public sector health-care providers on neonatal survival: a community-based cluster-randomised trial in rural Pakistan	Soufi et al.
Online participatory intervention to promote and support exclusive breastfeeding: randomized clinical trial	Cavalcanti et al.
Effect of home-based newborn care on neonatal and infant mortality: a cluster randomized trial in India.	Rasaily et al.

Training and evaluation of Community Health Workers (CHWs): towards improving maternal and newborn survival in an urban setting in KwaZulu-Natal, South Africa.	Ndaba et al.
Strengthening the Community Support Group to improve maternal and neonatal health seeking behaviors: a cluster-randomized controlled trial in Satkhira District, Bangladesh.	Gai Tobe et al.
Newborn care practices at home: effect of a hospital-based intervention in Sri Lanka.	Senarath et al.
Adaptation of kangaroo mother care for community-based application	Quasem et al.
Can mothers recognize neonatal illness correctly? Comparison of maternal report and assessment by community health workers in rural Bangladesh	Choi et al.
Introduction of newborn care within integrated community case management in Uganda	Kayemba et al.

Can a community health worker and a trained traditional birth attendant work as a team to deliver child health interventions in rural Zambia?	Yeboah-Antwi et al.
Effectiveness of the baby-friendly community initiative on exclusive breastfeeding in Kenya	Kimani-Murage et al.
Community-based father education intervention on breastfeeding practiceâ€"Results of a quasi-experimental study	Bich et al.
EMBRACE intervention to improve the continuum of care in maternal and newborn health in Ghana: The RE-AIM framework-based evaluation.	Kikuchi et al.

Scaling up Kangaroo Mother Care in Ethiopia and India: a multi-site implementation research study	Mony et al.
Effectiveness of a Quality Improvement Program Using Difference-in-Difference Analysis for Home Based Newborn Care - Results of a Community Intervention Trial.	Dhanesh Goel et al.
mHealth intervention “ImTeCHO” to improve delivery of maternal, neonatal, and child care services—A cluster-randomized trial in tribal areas of Gujarat, India	Modi et al.
LATCH Score at Discharge: A Predictor of Weight Gain and Exclusive Breastfeeding at 6 Weeks in Term Healthy Babies	Shah et al.
Telemonitoring of high-risk neonates discharged from SNCU using a novel device: a pilot study	Madireddy and Lingaldinna

Community health promotion and medical provision for neonatal health-CHAMPION cluster randomised trial in Nagarkurnool district, Telangana (formerly Andhra Pradesh), India	Boone et al.
Potential effectiveness of Community Health Strategy to promote exclusive breastfeeding in urban poor settings in Nairobi, Kenya: a quasi-experimental study	Kimani-Murage et al.
Effect of the Uganda Newborn Study on care-seeking and care practices: a cluster-randomised controlled trial	Waiswa et al.
Effect of implementation of Integrated Management of Neonatal and Childhood Illness (IMNCI) programme on neonatal and infant mortality: Cluster randomised controlled trial	Bhandari et al

Household surveillance of severe neonatal illness by community health workers in Mirzapur, Bangladesh: coverage and compliance with referral	Darmstadt et al.
Increasing access to care for sick newborns: evidence from the Ghana Newhints cluster-randomised controlled trial	Manu et al.
Effect of women's groups and volunteer peer counselling on rates of mortality, morbidity, and health behaviours in mothers and children in rural Malawi (MaiMwana): a factorial, cluster-randomised controlled trial	Lewycka et al.

Understanding how women's groups improve maternal and newborn health in Makwanpur, Nepal: a qualitative study	Morrison et al.
A community based approach to improve health care seeking for newborn danger signs in rural wardha, India	Dongre et al.
Community-based antenatal education in Istanbul, Turkey: Effects on health behaviours	Mulzan Turan and Say

Feasibility assessment of an ergonomic baby wrap for kangaroo mother care: A mixed methods study from Nepal	Thapa et al.
Linking Home Based New Born Care to the Existing Government Health system in Tamil Nadu - Pilot Study	Jeganathan et al.
Effect of community-based behaviour change management on neonatal mortality in Shivgarh, Uttar Pradesh, India: a cluster-randomised controlled trial	Kumar et al.
Key lessons from a mixed-method evaluation of a postnatal home visit programme in the humanitarian setting of Gaza	de Vries et al.

Explaining the impact of a women's group led community mobilisation intervention on maternal and newborn health outcomes: the Ekjut trial process evaluation	Rath et al.
Effect of Village Health Team home visits and mobile phone consultations on maternal and newborn care practices in Masindi and Kiryandongo, Uganda: a community-intervention trial	Ayiasi et al.
Men's knowledge and awareness of maternal, neonatal and child health care in rural Bangladesh: a comparative cross sectional study.	Nasreen et al.
Effectiveness of an integrated approach to reduce perinatal mortality: recent experiences from Matlab, Bangladesh.	Rahman et al.

Assessing community based improved maternal neonatal child survival (IMNCS) program in rural Bangladesh	Rahman et al.
Testing a scalable community-based approach to improve maternal and neonatal health in rural Nepal	Hodgins et al.
Effect of scaling up women's groups on birth outcomes in three rural districts in Bangladesh: a cluster-randomised controlled trial	Azad et al.
Effect of community-based promotion of exclusive breastfeeding on diarrhoeal illness and growth: a cluster randomised controlled trial	Bhandari et al
Comparison of the effect of two systems for the promotion of exclusive breastfeeding	Coutinho et al.

Exclusive breastfeeding promotion by peer counsellors in sub-Saharan Africa (PROMISE-EBF): a cluster-randomised trial	Tylleskar et al.
Can early postpartum home visits by trained community health workers improve breastfeeding of newborns?	Mannan et al.
Community-Based Kangaroo Mother Care to Prevent Neonatal and Infant Mortality: A Randomized, Controlled Cluster Trial	Sloan et al.
Postnatal peer counselling on exclusive breastfeeding of low-birthweight infants: A randomized, controlled trial	Agrasada et al.
Impact of an integrated nutrition and health programme on neonatal mortality in rural northern India	Baqui et al.

Education for expectant fathers in workplaces in Turkey	Sahip et al.
The Effect of Increased Coverage of Participatory Women's Groups on Neonatal Mortality in Bangladesh A Cluster Randomized Trial	Fottrell et al.
Effect of Postnatal Home Visits on Maternal/Infant Outcomes in Syria: A Randomized Controlled Trial	Bashour et al.
Improvement of perinatal and newborn care in rural Pakistan through community-based strategies: a cluster-randomised effectiveness trial	Bhutta et al.
Practice of skin-to-skin contact, exclusive breastfeeding and other newborn care interventions in Ethiopia following promotion by facility and community health workers: results from a prospective outcome evaluation	Callaghan-Koru et al.

Use of Job Aids to Improve Facility-Based Postnatal Counseling and Care in Rural Benin	Jennings et al.
Effect on Neonatal Mortality of Newborn Infection Management at Health Posts When Referral Is Not Possible: A Cluster-Randomized Trial in Rural Ethiopia	Degefie Hailegebriel et al.
Effect of an integrated community-based package for maternal and newborn care on feeding patterns during the first 12 weeks of life: a cluster-randomized trial in a South African township	Ijumba et al.
Can a community health worker administered postnatal checklist increase health-seeking behaviors and knowledge?: evidence from a randomized trial with a private maternity facility in Kiambu County, Kenya	McConnell et al.
Assessment of the uptake of neonatal and young infant referrals by community health workers to public health facilities in an urban informal settlement, KwaZulu-Natal, South Africa	Nsibande et al.

Effectiveness of conditional cash transfers (Afya credits incentive) to retain women in the continuum of care during pregnancy, birth and the postnatal period in Kenya: a cluster randomised trial	Vanhuyse et al.

**Type of intervention**
**Frequency/duration, mode**
Intermittent KMC was offered to mothers of late pre-term infants(34-36 weeks) in the postnatal ward. Healthcare workers were trained on the intervention and protocol, meetings were held with staff to ensure data was collected consistently and reliably. Healthcare workers educated the mothers pre-discharge. The study was conducted in 4 level 3 hospitals.
Immediate breast crawl was compared to standard care in a public hospital in Hyderabad. Mothers were randomly assigned their group. After birth immediate skin to skin contact was practiced, and the mother and baby were observed for one hour or until the baby first breastfeed, babies were assisted if they didn't crawl within 60 minutes. There was no skin to skin contact in the control group. Mothers in both groups were told the importance of breastfeeding and encouraged to feed on demand.
Community based management of LBW infants by female CHWs. CHWs receive 5 days of training and follow up on mothers for ANC, and newborn danger signs for LBW infants (once a week for 1 month). They can prescribe basic antibiotics for infection.
There were 2 intervention arms (home-based care and community-based care) and a control group. In the home care arm an NGO recruited one CHW per 4 villages and trained them for 6 weeks, which included skills development, behaviour change communication, provision of essential newborn care, clinical assessment of neonates and management of sick neonates using an algorithm from IMNIC. CHWs conducted surveillance to find pregnant women. They conducted 2 antenatal visits (birth and newborn care preparedness) and 3 postnatal visits. They classified sickness into very severe disease, possible severe disease (mroe than one sign) and possible severe diseas (one sign only). Severely ill babies were given one dose of an antibiotic and referred to a facility if they were sick, if they could not be referred they were given antibiotics at home. Those with one sign were referred, and a repeat visit was helf 24 hours later for monitoring and further referral.<br>In both intervention groups male and female community mobilisers held group meetings to disseminate birth and newborn care preparedness messages. In the community care arm female mobilsiers visited every 4 months and men visited every 10 months. TBAs encouraged women to attend these meetings. In the home care arm the female mobiliser visited every 8 months.<br>Government health workers in all 3 arms received refresher training sessions on management of maternal

Created maternal and neonatal health groups comprised of local healthcare staff and local key stakeholders (trained professionals, influential commune members). Faciliators were recruited from the Women's Union (social and national governmental org that works with issues around women's needs). Facilitators supported the group with monthly meetings. Women had to be members of the union, have secondary school education and have children. Selected facilitators had 10 days of training (theory, group discussions, role play). Group dynamics and quality improvement methods were the focus (brainstorming, nominal group technique, plan-do-study-act cycle, SWOT tool). They were also taught about evidence-based neonatal care and the health system. A facilitation manual was provided to help guide daily work. Facilitators practised their skills in rural communes out of the study areas and the study team provided feedback. Each facilitator worked with 5-8 groups.<br>The intervention was meant to train facilitators in participatory, problem-solving approaches, empower and support members to identify local problems and actions related to neonatal health and improve health outcomes for neonates. Previous studies showed the MNHG had a good mix of people, though progress may be slow.
VHWs were trained women with 5-10 years of education in intervention villages. VHWs registered pregnant women, usually in their 4th month (cultural calendars to estimate due date). VHWs attended the birth and worked out if the baby was pre-term using the calendar and recorded it on a printed mother-neonate record. The babies birth was measured max 24hrs after delivery. The scale was tested with standard weights once every 3 months. Babies which were pre-term, LBW or didn't feed on the first day received more care from VHWs. VHWs visited on days 2,3, 5, 7, 15, 21 and 28. High risk neonates had additional visits on days 4, 6, 9, 12, 18 and 24. Weight was measured every week for 28 days and charted, less than 300g weight gain was inadequate. VHWs could also make extra visits if the mother said the baby was ill. VHWs monitored until day 28, the mother and baby left the village or the baby died. Interventions included: health education for mothers (2hrs group education every 4 months, 45 mins individual twice during pregnancy and once after, pamphlets for high risk neonates), thermal care, breastfeeding, prevention and management of infections, management of neonatal sepsis, vitamin K injection, referral.<br>A supervisory physician made visits to the villages to check on neonates once every 15 days. Records were checked and corrected, findings and care checked. KMC was added, as was an evaluation form that
Participatory women's groups mobilise communities around maternal and newborn health. Followed an action cycle (identify and prioritise problems, decide upon local solutions, implement, and evaluate solutions). Max 37 women in a group, one group per 1200 people. Half the groups had maternal and neonatal health task forces (MNHTF) added to them by the program to identidy high-risk pregnant women and improve ANC/PNC coverage, knowledge and facility delivery. Groups came up with health education, voluntary testing and counselling for HIV/AIDS, village savings and loans, bednets, vegetable gardens and bicycle ambulances.<br>Quality improvement at facilities - plan-so-study-act cycles within hospitals with providers; change packages; death reviews; training.<br>Community surveillance system (volunteer key informants approved by village development/health committees) collected information on pregnancies, births and deaths of consenting women. KIs were

Pregnant women were identified by door to door screening, and were followed up more frequently closer to their due date. Babies weighing 1500-2250 were enrolled as soon as possible after birth, but not more than 3 days after birth. Babies with feeding and breathing problems excluded. Babies weighing 1500-1800 were referred to hospital per guidelines, but were allowed to participate if they didn't go to hospital or went and came back before 3 days.<br>Intervention group received community initiated KMC and control group receives normal care.<br>Intervention consisted of promotion and support of STS contact and EBF by intervention workers and supervisors. Counselled on preference for 24hr of STS care, during home visits care was observed and support given for any issues. Intervention workers were similar to ASHAs, and supervisors to ASHA supervisors. They were both trained on KMC and counselling in hospitals. Babies were visited on days 1-3, 5,
LHWs (lady health workers, CHWs) were trained to deliver preventive and promotive community mobilisation and an education package. They were trained on bag and mask resuscitation of asphyxiated newborns and recognition and management of LBW babies, neonatal infections and referring babies to public hospitals when needed. LHWs were linked with TBAs so that they could attend deliveries. LHWs were given clean birthing kits. LHWs were trained to make postnatal visits on days 3, 7, 14 and 28 after birth. TBAs were given 3 days basic training on immediate maternal and neonatal care. LHWs received compensation for transport to any visits.<br>LHW supervisors were trained for 5 days, they went on to train LHWs at the facility they are assigned to for 3 days with monthly refresher sessions. LHWs were given pictorial guides on resuscitation, thermal care, co-bedding, BF LBW babies, recognising pneumonia and administering amoxicillin.<br>Male CHWs received separate training on health education and community mobilisation. They focused on
The research team were trained for at least 20 hours on data collection procedures and logistics. Phone calls were made to participants for 6 months after they were discharged.<br>A booklet with info on breastfeeding was created by the team, based on the Brazilian MoH and WHO recommendations. Meassages were illustrated and easy to understand. Both intervention and control groups received the booklet. The intervention group had a closed Facebook group which ran for 24 weeks for each mother, content was based on the booklet and discussions in the group. Each week a subject from the booklet was posted, mothers were tagged and encouraged to comment their experiences and another topic that was important for them at the time. The team monitored the messages. Researchers answered
The HBNC intervention was either delivered by a specially recruited village health worker or an existing CHW. The specially trained health workers had to be residents in the village, educated up to a certain level, be a mother, willing to attend delivery at night and undergo residential training.<br>The specially recruited village workers and existing CHWs received the same training. The SEARCH team trained the field supervisors in workshops for 15-17 days. The supervisors trained the community workers for 5 days and field practice of 1 month. One set of skills were taught at a time via classroom sessions and hands-on training. In both intervention arms healthcare workers also received training. Reorientation and on-job training was undertaken during monthly meetings.<br>Surveillance was conducted to find pregnant women. The intervention consisted of health education, care at birth, care of normal/LBW neonates, identification and treatment of sick neonates and young infants and community mobilisation.<br>Health education consists of early initation and EBF, infection prevention, prevention and management of hypothermia, recognising danger signs and seeking immediate care from health workers. The CHWs tried to attend deliveries. Health education included a flipchart and DVD.<br>Healthy babies received 12 visits at home and high risk newborns received 17 visits. The CHWs assessed

The CHWs were already working in the 3 24/7 PHC that were selected (for having high workloads which affects quality). General CHW training covers 12 topics.<br>Training took 3 days and covered pre and post delivery. Utilisation of MCH services and newborn danger signs were included. The training was meant to enhance the relationship with the community advisory group and partner defined quality (planning and designing with the community,bridging the gap between facilities and community care). Lectures, discussion and role-plays were used. Pre post tests were given around training to understand knowledge retention, spot checks were done with mothers and shadow visits with
A community support system was set up so the community could organise themselves and implement activities to promote safe motherhood. Community diagnosis and resource mapping exercise, Advocacy and planning meetings at union level, Establishment of CSGs, Capacity building of CSG members and Union Parishad (local government) for implementing community mobilization activities, Promoting birth planning, ANC, PNC, and neonatal care counseling and timely referral through engaging selective female CSG members and mobilizing local resources, Maternal and perinatal death audit at the community level, and Enhancement of maternal and neonatal health-related knowledge and practices among the residents.<br>CSG members registered pregnant women, helped them with the CoC, birth planning, ANC and PNC and referred them to care where needed.
Hopsital health care providers (nurses, midwives and doctors) were trained for 4 days on ENC (essential newborn care). The health care providers were trained to educate mothers on caring their newborns before, during and after the delivery, specifically through a health education session before discharge from the hospital.<br>All HCWs in the obstetric unit of 2 hospitals participated. The training took 32 hours.
Developed a protocol for initiation of community-based KMC, which doesn't not require birth weight or clinical judgement to determine should receive KMC. Women and their support system were taught about CKMC during pregnancy with messages reinforced after delivery.<br>Person-to-person communication and demonstration of skin-to-skin contact was implemetned, as was: promoting the cleaning of babies with a damp or dry cloth; promotion of breastfeeding on demand; and advising to seek medical care for babies' danger signs. Laminated flash cards with pictures were used as education tools.<br>Community-based nutrition promoters were trained on KMC for a week, using hands-on methods and role plays. They had meetings to discuss their experiences teaching KMC to mothers.
CHWs had 36 days of training on the interventions and surveillance. Field supervisors monitored CHWs neonatal assessment and management at scheduled and random points during fieldwork. Biweekly refresher trainings were held. CHWs made two prenatal visits at home, at around 12–16 and 32–34 weeks of gestation, and promoted birth and newborn care preparedness, including recognition of 13 neonatal danger signs. Mothers, mothers-in-law and husbands were told they need to take their baby to a facility if it demonstrates one of the danger signs. CHWs used cards with pictures to describe the signs and left them with the families for reference. CHWs tried to attend births, or visit twice within the first week of birth. During postnatal visits CHWs conducted neonatal surveillance via a standardised assessment form. Mothers were asked if the baby had any of the 13 danger signs and CHWs conducted a physical assessment of the baby.<br>The 2 districts had differences in their algorithm, treatment (injectable antibiotics vs referral only) and where
iCCM (integrated community case management) is ongoing in Uganda - a neonatal component was added. VHTs (village health teams) made up of 5-6 volunteers in the community who deliver maternal, neonatal, sanitation and child health interventions. 2-3 members received training on iCCM.<br>VHTs are trained to make visits on day 1, 3 and 7 post-birth and refer sick newborns to health facilities with extra supportive care for small newborns. VHT members has 6 days of training for VHTs, and another 6 days on iCCM (2 hours on neonatal health). They had malaria and pneumonia medication in case children required it.

Existing CHWs and TBAs were paired, no teams created for places lacking a CHW or TBA. The pair were trained with a pair of NHC members on teaming concepts (specific tasks and skills/competencies). This incldued communication, respectful dialogue and action, mutual support and working together, making decisions and managing conflict, trust and confidentiality, team maintenance, evaluating success/failures, asking for feedback, motivating each other.<br>Tasks included: meeting NHCs, behaviour change communication, problem solving for newborn and child care, outreach services, support referral, intra-team referral, postnatal care visit at 6-8 weeks.<br>Training mentioned the importance of completing tasks and documentation. Training included exercises, demonstrations, role play, brainstorming and real-life scenarios.
ToT from the MoH trained the sub-county health management team, HCWs and CHEWs. The implementation package was developed by the MoH and adapted from the WHO/UNICEF integrated infant and young child feeding course. On the job training and mentoring occurred quarterly to ensure proper implementation of BFCI.<br>CHVs, TBAs and other providers at the community level received training, incluidng establishing and conducting mother-to-mother support groups. Each CHU was given $1000 to create an income generating project. CHVs were given counselling aids.<br>Women in the intervention group received at least 8 visits from trained CHVs. Visits occurred once pm upto 37 weeks, every 2 weeks until the first month postpartum, once pm until the baby is 6 months old. Women who had challenges feeding infants received more visits.<br>Support groups met monthly to discuss and learn about pregnancy, nutrition and other health issues. There
An integrated community-based educational intervention targeting fathers at antenatal, delivery, and postnatal periods for supporting breastfeeding practices in Vietnam. It's a community based intervention integrated into routine healthcare service provided by local staff. Messages were aired on loudspeakers twice per day, flyers, mugs, calendars, posters were all used.<br>Midwives and commune health workers were trained on breastfeeding in a 2 day workshop, followed by 3.5 months field training in a site that was not included in the study. Trainees received feedback from trainers and fathers while conducting their field training. the intervention package consisted of components including mass media communication; group health education and counselling; individual counselling; and social public activities.<br>Individual counselling happened at delivery and at home visits (last trimester, week 1, 6th week, 3.5 months).<br>Fathers were told about the benefits of BF and how to support their wives. They were given a calendar showing the development of infants until 1 year and how they could support them. Monthly meetings were held, but unexpected meetings could aslo be called for specific issues. A contest was held to see which father
The intervention aimed to reduce the coverage gap of PNC 2 days after birth, accelerate understanding of CoC amonst HCWs, encourage community members to support mothers in getting maternal and neonatal care from skilled providers.<br>CoC components defined as 4 ANC visits; delivery by skilled attendant; PNC at 48hrs, 7 days and 6 weeks.<br>The intervention consisted of use of CoC cards (1 page pictorial educational record card, used to exaplin the value of CoC and encourage women to receive continuous care. Attached to maternal health record book. Different coloured stars used depending on when care was received), CoC reorienting for HCWs (monthly support visits were held), 24 hour retention of women + newborns in the facility after delivery and PNC via home visits.<br>Program provided some equipment e.g., stethoscopes and thermometers, beds and motorcycles.

State and district level government health managers and a local research org were partnered and challenged to come up with an implementation strategy to reach 80% coverage of KMC. 1 - accurate birthweight recording and referral of babies from home/facilities that don't provide KMC; 2- changes in infrastructure and training/motivaion/support for facility staff; 3 - post discharge KMC continuity (links between facilities and CHWs, home visits, champions to help other mothers, communtiy events, performanc of CHWs reviewed in regular supervision).<br>Formative research showed scales that didn't work well, crowded conditions, HCWs who didn't use KMC even though it's in the national guidelines (thought incubators were better) and mothers who wanted to leave after 6-12 hours due to perceived lack of quality care. CHWs often failed to make home visis post discharge.
ASHAs (CHWs) received 2 days of training on technical and communication skills followed by supportive supervision in the field. Training included case studies, videos, live demonstrations, lectures, groups discussions, role plays. ASHAs were provided with technical and logistic support for community meetings and a gift pack including items for newborn care (cap, gown, towel etc.) were given to each mother. Health messages included ealy initiation of BF, EBF for 6 months, delayed bathing, KMC, hygiene, danger sign recognition and free ambulance service. Catchy slogans of key messages were painted in intervention villages.<br>Supervisors used a checklist to rate ASHAs conducting home visits and provided feedback after the sessions. Group meetings were used to disseminate health messages uisng posters and live demonstrations, followed by group discussions to clarify any messages.
mhealth intervention which consists of a variety of tech-based job aids for the different levels of HCWs involved in service.<br>ASHAs were given a mobile phone with a postpaid data plan. Women and children under the age of 2 were registered on Imtecho. The software prepared a schedule of home visits as per the national standards and sent reminders to ASHAs. A digital form was completed during the home visits. ASHAs showed short video clips with health messages to families during home visits. The digital forms had checklists to screen for complications and a decision support system with home remedies and contacts for emergency services. Immunisation was also recorded. Performance based incentives were calculated automatically on the app and disbursed. Each medical officer was given a tablet. The medical officers and PHC staff used the web interface to track high-risk cases, view reports, and manage incentives and supplies.
The LATCH score was determined after a 10 minute observation. L - latch, A - amount of audible swallowing, T - type of nipple, C - comfort during feeding, H- amount of help the mother needs to hold her infant to her breast. The score runs from 0-10, with higher scores being better. Two LATCH scores were taken, one within 24 hours of birth and another at discharge. Mothers with low LATCH scores at the first assessment were counselled and shown the correct technique by a lactation nurse to improve the score before discharge. At 6 weeks babies were weighed and the type of feeding was recorded. Babies were weighed daily at home after day 8 by ASHAs.
Home-based monitoring device that detects health indicators related to respiration, chest movement, crying, and activity level through video and audio accounts. Parents need to upload 4 short (2.5 min) videos of the neonate on a specific app, and trained health staff review them to assess the neonates condition. If nurses identify issues through the videos they connect the parents to ASHAs to support them in seeking further care, if necessary.<br>Parents were instructed on how to use CareCradle, it consisted of a baby mat and smartphone mounted on a plastic frame. Videos were automatically sent to clinicans. Nurses called parents twice a day to collect info on the baby's condition, feeding patterns and other relevant details. If nurses noticed anything abnormal videos were sent to consulting physicians. Physicians briefed the nurses, who advises parents on appropriate home

Community health promotion and provision of health services. Health education campaigns were held in the village, song and dance (folk culture) were used to promote key maternal and child health themes. Artists helped create the songs, which were piloted. The messages included: newborn care, misconceptions about MCH, danger signs for pregnanices etc.<br>PDGs were held monthly with women to improve knowledge and increase awareness of services available. Mothers could also discuss solutions to MCH issues. Health service use before and after delivery and danger signs were brought up repeatedly. The sessions lasted an hour in a convenient location for women. Flash cards, posters and flip charts were used. Ice breakers were conducted.<br>Women were selected after discussion with the community to be trained as VHWs. VHWs recruited eligible women, attended deliveries, made postnatal visits at home, tracked high risk mothers and referred mothers or newborns to facilities where needed. VHWs received some medical equipment and were paid. VHWs had one week of training followed by quarterly refresher sessions. Midwives received 10 days of training and accompanised VHWs and made regular checks on mothers and newborns in the area.<br>Private health facilities agreed to serve women from the intervention area at a subsidised fee, the team provided ambulances for referrals. Women had health cards to record services received.<br>Control area got ECL (ensuring children learn) intervention, so that the quality of data collected in
CHWs in the intervention arm were trained on maternal, infant and young child nutrition; CHWs in the control were trained on standard practice (antenatal and postnatal care). CHWs in both arms provided information, education and communication material to mothers (brightly coloured cards which show behaviours and concepts), and were compensated $35 per month. The intervention is delivered by cluster.<br>Timing of intervention: Home visits from CHWs once per month until 34 weeks of pregnancy, every week after until birth, once per month until the 5th month then fortnightly to prepare for mixed feeding, then once per month until 1 year.<br>Counselling covered: maternal nutrition, immediate initiation of breastfeeding after birth, breast positioning and attachment, EBF, frequency and duration of breastfeeding, expressing breast milk, storage, handling and feeding of expressed breast milk and lactation management and age-appropriate complementary feeding.
CHWs are trained for 5 days to make 2 home visits during pregnancy and 3 after birth (days 1, 3 and 7) to offer preventive and promotive care and counselling; small and sick babies get extra visits. Health facility strengthening was done. One CHW per 100-150 households.<br>VHTs are used - 5-6 volunteers who work on community mobilisation preventive health. 1-2 members now focus on MCH and newborn health.<br>There were quarterly supervision meetings and direct observed supervision.<br>CHWs only received a travel reimbursement for supervisory meetings, otherwise getting a t shirt, briefcase and certificate.
CHWs, nurses and physicians in the intervention areas were trained in improving case management skills using the government's IMNCI training modules. Community-based HCWs were trained in the IMNCI basic healthcare worker course. Trainers visited trainees at work and reviewed performance, helped overcome challenges to implementation and suport the use of skills learned. Private practitioners were also invited to participate in the training sessions.<br>Supervision of CHWs was strengthened in the intervention area. Task based incentives for IMNCI, including postnatal home visits, were included. Drug depots were established in villages to ensure regular supply of IMNCI drugs to CHWs treating newborns and older children.<br>CHWs made postnatal home visits on days 1, 3, and 7 to promote early and exclusive BF, delaying bathing, keeping the baby warm, cord care, and care seeking for illness. They assessed newborns for signs of illness and treated or referred them. LBW babies had extra visits on days 14, 21, 28. CHWs ran women's group

CHWs were trained for 35 days, including 6 days of field practice, on pregnancy surveillance, essential newborn care, routine neonatal assessment, and management of illness based on a clinical algorithm adapted from the Bangladesh Young Infant IMCI algorithm.<br>The intervention linked community level assessments by CHWs with outpatient or inpatient records at a local tertiary hospital. CHWs recruited pregnant women from routine surveillance and visited them twice antenatally to discuss birth and newborn care preparedness (BNCP). CHWs gave mothers a BNCP card and told them to bring it with them to any visits at the hospital; mothers were also given a labour card that should be presented to CHWs once labour started. CHWs tried to atend delivery or visit soon after. Postnatal home visits were planned for days 2, 5 and 8. During the visits CHWs completed a standardised newborn assessment form, identified serious illness and referred to the hospital as specified by the algorithm. Very severe disease was classed as having 8 signs, then changed to 11, and possible very severe disease was 9 signs, then changed to 6.<br>CHWs provided families a referral card and facilitated transportation, and all care at the hospital is free. CHWs managed neonates at home who did not comply and encouraged compliance. At the end of the firsy
Community based surveillance volunteers (CBSV) were trained for 9 days to make home visits (2 during pregnancy , days 1, 3 and 7 post-birth) and promote ENC practices, weigh babies and provide referrals for sick newborns. CBSVs left their contact with mothers so they could visit them on time post-delivery and increased frequency of visits for women in late pregnancy. CBSVs weighed newborns and looked out for danger signs, and referred when required; they counselled families on danger signs and emphasised the need for immediate action when newborns are ill; they spoke with families and problem-solved around barriers to care seeking. They counselled on the importance of saving money during pregnancy in case of an emergency. CBSVs has portable weighing scales, colour coded bands (red below 1.5kg, yellow 1.6-2.4, green over 2.5), thermometer and timer.<br>Training consisted of interactive discussion, groups discussions, practical WHO videos, and 2 days conducting clinical assessments in hospitals (at least 2 babies assessed).<br>Referral cards were provided and mothers counselled on the importance of keeping babies warm and breastfed during transit. Check-ups happened 24 hours later, if babies weren't referred they were re-assessed.
There was a cluster facilitator (local mothers, literate, selected from the communtiy), they did an action cycle of 20 meetings in 4 phases. 11 days of training with refreshers every 4 months. Facilitators received a salary, bicycle, umbrella, field bag, forms. Facilitators had supervisors visit them twice a month for observation, training and feedback.<br>Facilitators used a manual to implement the cycle, picture cards of maternal and newborn health issues were used to guide discussions. 4 phases: identify and prioritise MCH issues; identify strategies to implement; plan and implement; assess and make plans for the future. Groups decided membership criteria,, in phase 3 men were allowed to join.<br>Volunteer peer counsellors selected from the community. Made one visit during pregnancy and 4 after birth (1 week, 1 month, 3 months, 5 months). Education on EBF, infant care, immunisation, PMTCT, FP. Supported those with breast issues and encouraged care-seeking. Intervention manual (visit content) and picture book used. 5 day training and annual refresher. Bicycle, meeting allowance, calendars and forms provided.

Qualitative evaluation of a participatory learning action cycle conducted by women's groups in Nepal. The groups were led by local women and met once a month. Issues affecting health were discussed, then problems identified, planning and participatory evaluation of solutions.
Community mobilisation and health education for families with sick newborns. Surveys and FGDs were conducted to learn about knowledge around newborn danger signs and understand healthcare seeking practices. Based on formative research and quantitative data, program staff developed simple health messages including local words for newborn danger signs and pictures appropriate to local rural context. Community based orgs (adolescent group, farming group, village coordination committee - VCC) were set up for community level action. VCCs raised funds for community level action, VCC members had capacity building during their monthly meetings to become gender sensitive with culturally appropriate solutions to local problems and develop emergency transport plan. Female CHWs were trained in identification of newborn danger signs and prompt referrals, they were supervised by local VCCs.<br>Monthly education sessions on maternal and child health - including neonatal danger signs and where to seek care - were covered by a social workers, auxiliary nurse midwife, VCC members and CHW. CHWs conducted home visits after delivery and government healthcare providers were given skills-based training.
The education sessions consisted of 2 sessions (2 hours) per week for one month at a community centre. Each session was led by a nurse, a facilitator and a trained community member. The program was free and completing 7 sessions gave mothers a certificate and a small gift.<br>Session topics included health during pregnancy, pregnancy nutrition, preparing for childbirth, childbirth, motherhood and communication, infant feeding, infant care and health, women’s health and contraception after the birth. The education programme was specifically designed for a population that has received little or no sex and reproductive health education as a part of formal education or otherwise.<br>Neonatal components included common illnesses, BF, bathing, dressing etc.

HCWs at the hospitals received 2 days KMC training based on national guidelines. HCWs identified mothers with stable LBW babies in hospitals. Mothers were interviewed over the phone every week for 28 days and returned for a postnatal visit. Mothers were split into 2 groups, the first received the new wrap followed by the traditional wrap, the second group received the traditional wrap first followed by the new wrap. HCWs counselled consenting mothers and their family on on the benefits of KMC and demonstrated the steps of correct KMC with both wraps. Families practices KMC with both wraps on a dummy, once they were comfortable they tried it on the baby for 6 hours then switched to the second wrap for 6 hours. They were offered one of the wraps for free to continue KMC at the facility and at home. Mothers were encouraged to stay at the facility for 3 days to monitor for sepsis. The traditional wrap is made of thick and warm flannel cloth, mothers ned help tying and untying it. The new wrap is ergonomic and can be tied and untied without help.
The intervention aimed to integrate home-based neonatal care (HBNC) into the existing health system, with referral linkage to NICUs. The existing system included integrated management of neonatal childhood illness (IMNCI).<br>NGOs assisted with implementation of the HBNC package and filled in the gaps in health facilities, mostly through provision of nurses for the NICU and maintenance of equipment.<br>HCWs were trained on IMNCI, CHWs referred cases (no provision of medication), 2 ambulances were used for neonatal transport specifically and a separate phone was attached to the out born unit for CHWs to call in case of any issues. The HBNC coordinator was the link between CHWs and the hospital, they would arrange admission and inform the CHW when the baby was discharged and advise on follow-up. CHWs advises these
There were 2 intervention arms - one received a package of preventive essential newborn care, including skin-to-skin care between the infant and a family member, promoted through behaviour change management, layered on existing services available to the control group. The second intervention group received the same but in addition had a crystal sticker which indicated hypothermia by changing colour.<br>The intervention package consisted of essential newborn care: birth preparedness, hygienic delivery, and immediate newborn care including clean umbilical cord and skin care, thermal care including skin-to-skin care, breastfeeding, and care-seeking from trained providers.<br>The intervention included home visits by volunteers, community meetings and folk song meetings. CHWs were trained for 7 days on KAPs and behaviour change, and were then monitored by a supervisor before being selected for the program. Mothers who did well were promoted as role models to other pregnant
Midwives and nurses were trained on the<br>national postnatal care protocol including home-based postnatal care, neonatal care, postpartum complications, behavioural change communication, and early childhood development. They were given kits for home visits and conducted the first visit within 48-72 hours post-discharge. The next 2 visits (after 1 week and after 6 weeks) could be conducted at home if necessary.

Participatory learning and action cycle in 244 groups over 3 years, which included 20 meetings. The groups consisted of 15-20 people and met monthly to discuss issues related to pregnancy, childbirth and the postnatal period. Facilitators received basic health education and training on participatory communication methods. Group members had meetings with the wider community to get their support in implementing strategies for pregnancy and childbirth. 4 phases - identify and prioritise problems (introduce the project and women's group cycle, identify maternal problems, identify neonatal problems etc.), plan strategies (discuss causes and solutions, identify strategies,opportunities and barriers), implement strategies (discuss strategy, review progress, discuss home care and facility care soltuiosn for the problems), assess impact (discuss activities and achievements, discuss possible behavioural changes in the community).
VHTS received training on making home visits to disseminate essential maternal and newborn care. They were given a mobile phone to make regular consultations with HCWs at facilities for clarification/advice. HCWs are also given a mobile phone. Voice communication was preferred over SMS as it allowed interactive consultation.<br>48 VHTs were trained for 5 days and were reimbursed $5 per month for transport. 14-17 participants - preferance for smaller groups. Training was based on the VHT handbook. Role plays and practicals were involved. Social science researchers would randomly sit in on home visits to make sure the discussions were on topic and pregnant women were interviewed. 2 refresher sessions were held.
Areas were split into control (no intervention), partial intervention (implementation for 6 months) and intervention (implementation for 2 years). Birth planning (where, saving money) discussions were conducted with mothers, fathers and other members of the family. MNCH committees consisting of 9-11 elite people (teachers, religious leaders, village doctor) were formed to discuss important MNCH issues. The committees monitored and facilitated delivery of MNCH services at the community leve, arrange financing, support referral of complicated cases, and arrange transport for referral and audit deaths. The meetings were devised to improve the involvement of men in MNCH services.
An integrated maternal and newborn health program using community- and facility-based approaches on perinatal mortality in a rural area in Bangladesh.<br>The MNCH program followed a continuum of care approach from pregnancy to delivery to postnatal improving links between community and facility based services.<br>CHWs identified pregnant women, made ANC visits and postnatal visits on day 1 after home delivery, and days 3, 7 and 28 for all women. CHWs referred babies if they recognised danger signs using an algorithm based on WHO guidelines, they provided counselling on well-being and BF. HCWs received training on complicatins, including resuscitation. A KMC unit was constructed fro LBW or premature babies. A checklist was used for quality insurance, whenver appropriate care wasn't provided checks were conducted until the

The intervention package was designed to address bottlenecks on demand and supply to ensure a continuum of care from home to the hospital. Community health workers were used to implement the program. The interventon aimed to increase knowledge and practices related to maternal and neonatal health, improve provision of quality services at the household and community level, increase availiability and access to quality services at facilities and increase participation, accountabiltiy and responsiveness to communities' voice in health services. Women received the intervention from the beginning of pregnancy until the child was 5. Program organisers and managers trained the community health workers. The CHWs provided care for LBW babies and referals to babies who were sick (birth asphyxia, sepsis, respiratory infections). They visit
Birth preparedness package (antenatal health education package) - covered essential newborn care. Female CHVs provide antenatal counselling to pregnant women and families (husbands and mothers-in-law), this included giving a pictorial handout. The content focused on care seeking (delivery, ANC) and response to danger signs (individual planning and practical support) and household practices during pregnancy and essential newborn care (temperature control, hygiene and BF). Female CHVs dispense during antenatal visits and postnatal visits and make a postnatal visit within 3 days of birth where they reinforce counselling, assess
Both areas received health system strengthening (TBAs received basic training). Intervention areas had community-based women's participatory groups. Community leaders were approached for permissiong to establish these groups. Orientation meetings with union leaders and village mapping exercises (location of health facilities and social/religious places of meeting).<br>Women's group facilitators visited every 10th house and invited married women of reproductive age to join the group. Requests were made for adolescents and mothers-in-law to join, and they did later on. Local peer facilitators were used, they handles 18 groups, and received 5 training sessions on maternal and neonatal health issues and participatory modes of communication. Facilitators supported the group. Local supervisors supported facilitators with meetings with community leaders. Participatory learning action cycles were used (identify and prioritise difficulties, plan strategies, put strategies in place, assess effect). Meetings were held once a month for 20 meetings.<br>In the TBA intervention clusters receives basic training on clean deliveries, clean delivery kits and neonatal resuscitation. Control clusters did not receive the resucitation training.<br>Health service inputs focused on referral systems, links between the community and health services and
TBAs, local village-based workers, and auxiliary nurse midwives were used to counsel mothers. Messages included immediate breastfeeding after birth, feeding only breastmilk for the first 6 months of life, and breastfeeding the infant day and night, at least eight times in 24 h. Non-milk items which were commonly used were discouraged. Posters, flip books, a card, counselling guides were handed out at antenatal visits or home visits.<br>Health and nutrition workers received 3 days of training, with a hands-on component. Training included communication, detecting problems, and resolving difficulties. At each counselling contact the health worker assessed feeding practices, identified difficulties and provided info on the benefits of EBF.
There were 2 intervention groups - one where the BFHI content was used to train maternity staff, and the second with BFHI and 10 postnatal home visits (day 3, 7, 15 and 30; every 2 weeks in the second month and once a month during months 3-6) to encourage EBF.<br>HCWs at the facilities received 20 hours of training. Doctors were invited but didn't participate.<br>Copies of the UNICEF norms and routines for the encouragement of BF were offered to the hospital managers, together with posters, educational folders for mothers, and a MoH videotape on lactation management. Two copies of the book "Helping mothers to breastfeed" were given to each maternity hospital.<br>CHWs were recruited for the study, they received the same training as the HCWs and 5 days training and discussion on "helping mothers BF".<br>A booklet was used as the basis for discussion during home visits. Other family member's attitude to EBF was

One antenatal peer counselling visit on EBF, and 4 postnatal visits by trained peers. The data collection team were masked RE intervention/control clusters. Mothers were visited at 9-18 weeks for a week 12 target, and 18-28 weeks for 24 weeks.<br>In SA peer counsellors visited the control mothers at the same time points to help with getting birth certificates and social welfare.<br>3 attempts were made to make a home visit on schedule. Mothers who missed visits were revisited until the last scheduled visit.<br>Peer counsellors live in or near the intervention village and were trained for 1 week, they offer home-based-breastfeeding support. Fortnightly-mothly mentoring was provided. Referrals to health providers were given
The intervention consisted of trained CHWs doing 2 ANC home visits and 3 postpartum home visits (day 1-3, 4-5 and 6-7) to support and promote practices for birth and newborn preparedness and newborn care, including BF. CHWs conducted routine surveillance to identify pregnant women.<br>Postpartum visits focused on immediate newborn care, promotion and support for breastfeeding (positioning, attachment, sucking), recognition of newborn danger signs, care seeking and management of newborn infections, including referral.<br>CHWs were supervised for 2 days each month, and feedback was provided in monthly meetings. CHWs recorded BF status of each mother according to a standardised form.<br>CHWs received 21 days of training on BF, including lessons and practical sessions. Refresher training was
CHWs were trained for to teach CKMC to expectant mothers and their families. CHWs were compensated for their time, the equivalent of what they would be paid in nutrition programs. Monthly refresher trainings were held. Quarterly surveys identified pregnant women who wre recruited to the study. Babies were weighed within the first week of birth and followed up to just after their 1st birthday.
Mothers could receive breastfeeding counselling, childcare counselling or be in a control group with no counselling. VHWs participated in a postnatal care seminar at the hospital. Selected VHWs were given counselling training, they had some formal education and were willing to make home visits. All breastfeeding counsellors had previous positive experience with breastfeeding.<br>Counselling courses consisted of 40hrs of teaching, role-play and practical training. A certified lactation counsellor and a maternal child healthcare specialist trained the respective counsellors and monitored them throughout the study. Breastfeeding counsellors informed mothers of the benefits of EBF for 6 months and assisted mothers to prevent and manage breastfeeding issues. Childcare counsellors helped mothers with infant care and increasing mother-infant interaction via massages and smile therapy. 8 home visits, infant days 3-5, 7-10, 21, 1.5 mo, monthly to 5.5mo. The 2 groups of counsellors were unaware of each other. Peer
CARE-India, the government and local NGOs were all involved in the intervention. The newborn care package aimed to increase the frequency of behaviours that are proven to have benefits during antenatal, delivery and postnatal periods. The intervention encouraged anganwadi workers to recruit community volunteers (change agents). Anganwadi workers, change agents, and auxiliary nurse midwives received 6 days of training on the care of mothers and newborn babies. Information to encourage behaviour change was usually communicated during antenatal and postnatal home visits by these community-based workers. During the visit at 0-27 days postnatal counselling was provided on breastfeeding, essential newborn care (thermal care, hygiene, clean cord care), maternal and newborn danger signs and health-care utilization; follow-up visits for

Physicians attached to companies taught men about health and nutrition during pregnancy antenatal care, support of women during childbirth, breast-feeding, postpartum and postnatal check-ups, postpartum contraception, communication techniques and adjustment to fatherhood. Sessions lasted 3-4 hours. Follow-up surveys were done with fathers at 3 months and 9 months after birth. Focus groups were conducted with wives.
Intervention clusters had old groups from a previous study, who continued to meet once a month and and expand beyond maternal and neonatal health to discuss women and child health. The new groups discussed maternal and neonatal health. They used a participatory learning and action cycle. The coverage of groups was one per 309 people, higher than the previous study in Bangladesh. All clusters received health system strengthening - basic medical equipment, TBA and physician training and creating links between communities and health services.
Home visits were conducted by midwives to educate, support and counsel mothers on. Midwives had 5 days of special training, which consisted of a review of postnatal care, the role of home visits, the content of each visit, a physical exam, educational messages and communication skills. Group A received visits on days 1, 3, 7 and 30 after delivery, Group B received a visit on day 3 and C received standard care. Outcomes were assessed at 4 months.
The intervention focused on preventive and promotive care and was created in conjunction with the Sindh government. LHWs and TBAs were trained, liason between them was promoted and voluntary CHCs were created to promote maternal and newborn care in the village.<br>LHWs were encouraged to meet every pregnant women, visit them antenatally and on days 3, 7, 14 and 28 after delivery. TBAs were reimbursed for training costs but were not paid for their work, they also received training on resuscitation. CHCs did advocacy with elders and politicians, and were encouraged to organise an emergency transport fund and a use of vehicles using local resources. CHCs conducted group education sessions. Flipcharts and a docudrama were used as education tools.
Integrated package of facility based KMC and community based promotion of EBF and immediate SSC for all babies. HCWs at the facilities were trained on essential newborn care and the facilities were set up for KMC. HEWs were trained to counsel mothers on the importance of SSC and EBF and danger signs requiring immediate care seeking. HEWs were expected to make 4 ANC visits and 3 postnatal visits - 24hrs, day 3 and day 7. HDA (health development army) received training to support women in the community who had recently given birth - thermal care, immediate drying, SSC, EBF.

Nurse-midwifes were assisted with job aids, which reminded providers to counsel on immediate newborn care practices during the in-hospital period and at home, and recognition of danger signs for the mother and newborn.<br>Mothers started receiving messages during the first hour after birth, and recevied staggered messages throughout their stay in hospital so as not to overwhelm mothers.
Female volunteers from the community were selected to make things easier for the HEWs. Female volunteers received 4 days of training on counseling families about the importance of ANC, danger signs that should prompt care-seeking, birth preparedness, clean delivery, healthy newborn care practices to prevent infection, and how to indetify and refer sick newborns. HEWs received the same training and 3 days on volunteer support and 6 days on iCCM, 1 day on admin for the project - including how to inject kids. HEWs supported 10-15 volunteers, who each visited 20-50 households.<br>Volunteers visited twice during pregnancy, HEWs once; postnatal - volunteers visited on birth, day 3, day 7, HEWs within 2 days of birth and day 4. Volunteers and HEWs used pictorial material from the national family health card during visits.<br>Health centres were provided job aids and antibiotics, and healthcare providers received IMNCI training using the national curriculum (7 days).
CHWs delivered information via home visits to expectant and postnatal women.<br>In the intervention group CHWs were trained for 10 days on community entry, breastfeeding, danger signs, postnatal depression etc. Role plays, demonstrations and discussions were held. In the control group, CHWs were trained on documents and information needed to access social welfare. All CHWs were trained on how to use mobiles to enter data. CHWs were paid $300 per month.<br>Schedule: intervention - 2 antenatal visits, 48hrs after delivery, day 4, day 14, week 3-4 and 8-9. LBW received 2 extra visits in the first week.<br>CHW supervisors contacted CHWs when a birth was recorded in the hospital; all visits were attempted regardless of attendance. Control - 1 antenatal visit, 2 postnatal visits (week 6 and 12).
Postnatal check-ups are conducted either in-person by CHWs using a checklist on maternal and neonatal danger signs or via phone calls using the same checklist; these groups are compared to a standard of care group. Targeted health education is offered by the checklist groups. If danger signs were detected the mother was referred to the nearest facility. A nurse called the day after to check if appropriate care had been received.<br>CHWs are from the wider area, supervised by CHEWs and had received basic training. They were trained by the program manager and shadowed nurses on home visits and postnatal health education counselling.<br>CHWs received 4 days of training including role-plays. They were evaluated pre and post - clinicians signed
CHWs visit pregnant women in their last trimester and early postnatal period to deliver a packagae of interventions related to maternal and child health. 5 PNC visits (24-48hrs, day 3/4, day 10-14, 3-4 weeks, after 6 weeks). Extra visits for LBW babies. Mothers counselled on danger signs and referred to facilities where needed. CHWs wrote referral notes to local PHCs.<br>CHWs were trained and has metoring meetings every week.

CCT of $4.5 for attendance to ANC, facility delivery and PNC. The control group received $0.5 for airtime.
Card readers were used at the facility to enable automatic cash transfers.
Nurses were given $4 for each women enrolled in the trail and $1 for each woman at the end of the trial, payments were done electronically.

Intervention
**Success**
**How authors measured success, pros + cons, what they found**
EBF pre-discharge and 42 days post-discharge was measured. Mothers in the KMC groups were twice as likely to provide EBF at discharge (OR = 2.15) and EBF at follow-up (OR = 2.55). Older mothers and mothers with complications were less likely to provide exclusive breastfeeding.
Breatfeeding at 6 months was the main outcome - this was the same between groups, as it was at 6 weeks. Infant weight was significantly better in the crawl group at 6 months, as were maternal perceptions of breastfeeding and satisfaction with breastfeeding (this was at discharge).
NMR at 60 days was lower for the group with at least 1 visit than the group without a visit, as was the relative risk of death.
In the home care and community care arms te number of ANC and postnatal visits increased. Clean cord cutting, delayed first bathing and BF within an hour increased in thecommunity care and home visit arms, with higher increases in the home visit arms. NMR in the home care arm decreased by 1/3. CHWs referred over 1/3 of babies and treated more than that at home.

Facilitators were assessed on their skills, authors divided the clusters into "high" and "low" ratings for facilitators.<br>Monthly meetings were key to clarify the facilitators role and discussing the facilitation process and reporting on outcomes. Information on neonatal health and clinical practices was often requested in this meeting as facilitators thought this would be most helpful.<br>Brainstormin and plan-do-study-act cycles were most commonly used. Sometimes facilitators checked whether the intervention was executed with the desired effect - asking members if they understood and appreciated the messages delivered.<br>95% of planned meetings were completed. MNHGs participated for an average of 31 months. Groups identified 32 types of problem and 39 actions, they did this more in the first year than in following years. The most frequent issues were - low number of ANC visits, lack of PNC visits, low awareness amongst pregnant women of rest/diet/breastfeeding practices, home deliveries. The first year focused on pregnant women, with neonates brought in from the second year.<br>Actions taken mostly concerned dissemination of info in diverse ways/forums - counselling and mobilising women at home, communication at community meetings, counselling women at community health centres, communicating messags through village loudspeakers. Health information messages were created i the group, mostly by midwives.<br>In both low and high groups the NMR was lower than control, but the high group had a significantly lower OR of neonatal mortality.
Case fatality was the main outcome measure. 93% of babies born received HBNC. Coverage of the indicators was high for everything, except handwashing for mothers, KMC and referrals. Incidence of LBW decreased significantly, mostly due to intrauterine growth restriction decreasing. Case fatality for pre-term and LBW neonates decreased drastically, across all categories (gestation time, ranges of birth weights). The decrease in case fatality was accompanied by a decrease in the incidence of comorbidities. Sepsis, asphyxia and hypothermia decreased for LBW and pre-term babies; feeding problems decreased in LBW alone. Babies with sepsis benefitted from antibiotics, those without improved with supportive care alone.
Maternal, neonatal, and perinatal mortality were the primary outcomes. Clusters with both interventions of CI had lower stillbirths and perinatal deaths, with the effects being higher in the later months (higher dose). The combined intervention led to a reduction (22%) in NMR compared to control, reductions in asphyxia, prematurity and sepsis. Perinatal mortality reduced in the CI alone (stillbirths, sepsis, prematurity and asphyxia), neonatal mortality reduced in QI alone.<br>CI had a greater effect on perinatal and early neonatal mortality, FI on late neonatal mortality.

Authors were interested in mortality, EBF, measurements (weight, height), infection, diarrhoea, pneumonia.<br>Nearly all mothers in the intervention group gave KMC, while very few did in the control. KMC was given for longer in the intervention group too. More deaths occured in the control group, both in the first 28 days and at 180 days. EBF was more common in the intervention for the first 6 months. Weight and height were also higher in the intervention group at 28 days and 90 days. Growth effects were not seen at 180 days. No difference between the groups in hospitalisation or local infection, but the intervention groups had less diarrhoea and PSBI.
LHWs conducted most of the planned community education sessions. There was no difference between intervention and control when it comes to facility deliveries or births attended by a skilled attendant. A higher proportion of home deliveries were attended by LHWs in the intervention, and clean birth kits were used more in the intervention. NMR was lower in the intervention group than in the control. Breastfeeding within 1 h of birth, giving colostrum to neonates, and co-bedding or swaddling were significantly more common in intervention than in control clusters. Improvements in neonatal outcomes couldn't be ascribed to individual components of the intervention (resuscitation or antibiotics).
EBF rates were higher throughout the study compared to the control group. The median number of days of EBF was higher in the intervention group than in the control.
In the group with specially recruited CHWs a higher proportion of home deliveries were attended by HCWs, more newborns were examined 24 hours after delivery and more infants were suspected to have sepsis. NMR was lower in the clusters with specially recruited CHWs. Early neonatal mortality, young infant mortality and infant mortality were all lower in this group than the control. These rates were the same in the clusters with the standard CHWs and the control.<br>Rates of home visits and postnatal visits within 24 hours after birth were higher in the clusters with specially recruited workers than in the areas with standard CHWs.<br>The reduced NMR was seen regardless of whether deliveries happened at facilities.

Post-test scores averaged 63%; 67% was the average score from shadow visit - CHWs had good communication. The mothers rated them at 92% on average, showing that CHWs are trusted by the mothers and they have good information on newborn care.
Home deliveries decreased in the intervention group, and delivery in prviate facilities increased. ANC, PNC provided 42 days after delivery and care for sick newborns (all from skilled professionals) increased.
Compared to the pre-intervention, post-intervention mothers were satisfied with the health advice on caring for the newborn and were able to clarify any doubts with the HCW, i.e., they were more satisfied with the care provided.<br>Mother's knowledge on care of the umbilical cord and practices around this (e.g., not putitng spirit on the cord or covering the cord in a bandage) improved. BF was already practiced by over 90% of women pre-intervention, this rate remained the same post-intervention. The rate of undesirable health events since being taken home decreased in the post-intervention group.
The KMC messages were spread to the mothers, with high uptake of the flyers. All mothers had multiple contact sessions with the CNPs prior to delivery. All mothers had high levels of skin-to-skin care, this was higher in LBW and those with girls. Receiving info on KMC before delivery or 1 week after did not affect the rates of KMC, but KMC was initiated earlier if mothers had information before delivery. The rate of KMC dropped as time went on, starting at 66% in the first 2 days of life and ending with26% in the first month. BF was initiated early, but EBF for the neonatal period was low (extra fluids are usually given).<br>Interviews revealed that mothers had taken onboard a lof of the KMC information, but faced challenges around support, the discomfort of sleeping upright and conceptions of babies becoming stinky if only wiped with a wet cloth.
CHWs had high validity when compared to doctor's when it came to classifcation of VSD. Babies received 2-3 visits, depending which district they were born in. Mothers reported illness about half the times that CHWs reported illness. Maternal reports had poor sensitivity.<br>Maternal recognition did not vary by exposure to prenatal BNCP visits - interventions probably did not change maternal recognition of neonatal illness.<br>Compliance with referrals was low, especially for younger neonates. However, in both sites care seeking with a qualified provider was higher in the intervention than in the control group.
Knowledge/implemengint newborn care properly was the main succcess measure. The VHTs had good knowledge on newborn care, though they could not always list major danger signs (e.g., lack of breastfeeding). They referred sick newborns to facilities, and both them and HCWs thought carers listened to referrals.2/3 get direct feedback from HCWs on referrals, but only 1/3 follow-up with carers.

Measured teamwork and taswork via assessments, sums of the measures and averages were taken. 21 teams categorised as high achievers, 12 as low and 14 as inactive. CHW departure for a new job was the reason for the inactivity.<br>Mutual trust was high within teams, decision making/planning and mutual performance monitoring was lacking. Only 6 conflicts and these were all resolved well. Intra-team referral and joined problem solving were low. Community outreach and and education were joint activities that were well attended. Team scores on communication were high.<br>The most commonly recorded task is the handover at 6-8 week postnatal check up.<br>Teams that lived within an hour of each other were more likely to do well.
At 3-6 months babies in the intervention group are more likely to be EBF than children in the control group, but the authors said this wasn't signficant, at 2 months the rates of EBF were roughly the same. Mothers in the intervention group had a 3 times higher odds of EBF for 6 months than those in the control. Children in the intervention group were breastfed for longer (~21 days) than control.
Early initiation of BF (1 hour after birth) was higher in the intervention group, EBF remained higher in the intervention than in the control group at 1 month, 4 months and 6 months. Mothers in the intervention group were 1.7 times more likely to initiate EBF. Mothers in the intervention group were more likely to initiate EBF at 1 month and 4 months, at 6 months no mothers in the control group were breastfeeding, making a comparison difficult.
Reach, effectiveness, adoption (proportion of agents willing to implement a program), implementation (delivered as intended), maintenance (insitutionalised at organisational level).<br>Nearly 3/4 of the women in intervention had the CoC card, nearly 1/4 in control too; The number of women staying in the facility for 24hrs post-birth and receiving PNC within 48hrs after birth increased in intervention. The proportion of women completing CoC was higher in intervention than control. Proportion of newborns with danger signs was lower in intervention than control and a smaller proportion had complications at 6 weeks. KIIs said this was due to PNC home visits. Adoption of the interventions were high, minus the region where facility deliveries were difficult. HCWs said PNC at home was particularly well received as culturally women are not meant to leave the house 40 days after delivery. These visits could be difficult in regions where houses were far apart. Queues for examinations were reduced.<br>Implementation issues: HCWs leaving and not training new staff on cards; card stockouts; lack of 24hr stays due to rennovations/lack of sanitary facilities; difficulty riding bikes.

Coverage of STS care for more than 8 hours in the last 24 hours and EBF at hospital discharge and 7 days post-discharge were the outcome measures.<br>Coverage was 25% after model 1, 40% after model 2, close to 55% with model 3.<br>KMC was initiated for 82% eligible infants overall, 52% continued to receive KMC after discharge.<br>UP and Haryana had home visits by CHWs and and a call centre in UP - played a role in sustaining support at home.<br>Common elements for success: leadership and engagement of the governmet encouraged supervision of HCWs and financial resources for the large population being covered; convincing HCWs about the benefits of KMC and creating dedicated space ; and resources for mothers; systems for accountability and quality assurance (difficult); engagement of mothers; implementation science framework enabled networking between different groups.
ASHA's knowledge of neonatal care improved. Community meetings included grandmothers, fathers and newly married women. The majority of ASHAs greeted family members and built rapport with them. More than 1/3 of ASHAs didn't engage with mothers in a conversation about problems related to newborn care. Few asked about and addressed specific sociocultural issues. 1/3 did not counsel on cord care or danger signs. 1/3 of newborns were not examined for danger signs. ASHAs in intervention areas were more likely to make at least four home-visits, advice on cord-care, first bath and recognition of danger signs as compared to standard care group. Mothers in the intervention group were more satisfied with the communication skills and work of the AHSAs. EBF and prelacteal feeding didn't change much, but discarding colustrum decreased. ASHAs in the intervention group were more likely to conduct 4 home visits.
Uptake and adherence to the Imtecho app by ASHAs was good, however, PHC staff and medical officers did not use the platform as much as expected. The proportion of neonates visited twice in the first week and the modified ASHA-centric composite coverage index (MACCI) was significantly higher in the intervention than in the control.<br>Exclusive breastfeeding, home visitations during antenatal period, satisfactory counseling during antenatal period, early initiation of breastfeeding, ASHAs’ visitation at home within 24 hours of delivery (in case of home delivery) or within 24 hours of return to home from hospital in case of hospital delivery, satisfactory counseling during postnatal period, home visitations during postnatal period, newborn examination during home visits, and administration of ORS in case of diarrhea were significantly higher in the intervention clusters compared to the control clusters. Care-seeking for antenatal and neonatal complications was also significantly higher in the intervention clusters compared to the control clusters.
Median time for first feed was 2.5 hours and about 70% of babies were on EBF at 6 weeks. Having a LATCH score over 6 at the first test was an indication of EBF at 6 weeks. Having LATCH score over 6 at discharge was indicative of appropriate weight gain. Mothers who had an initial LATCH score under 6 were counselled and ended up with statistically significant higher LATCH scores. Prior experience with BF and counselling were both important factors for high LATCH scores. Infants with low LATCH scores at discharge were more likely to get hospitalised within the first 6 weeks post-discharge.
Utility and feasibility of CareCradle to identify early signs of illnesses in neonates discharged from SNCU (sick neonatal care unit).<br>Only half the vidoes requested were sent (mothers busy with house work, network issues, neonate seemed uncomfortable while recording video), but about 80% of audio calls were successfully completed. Videos allowed most health indicators to be evaluated at a high rate, except respiratory rate and quality of cry. 2 parents received counselling on propoer feeding techniques, and 2 neonates were referred to healthcare facilities. One of these babies was treated for pneumonia. Another baby died (only on video sent, the baby was brought to hospital but no sign of life).

NMR was the primary outcome. Secondary outcomes included cause of neonatal death, maternal mortality rate, health knowledge, health service usage and satisfaction with care.<br>NMR was slightly higher in the control group than in the intervention. Women in the intervention group could recognise more risk symptoms for neonatal problems than those in the control group. Women in the intervention group were more likely to have attended more antenatal care, with more testing and advice, delivery at a facility, delivery with trained personnel, antiseptic dressing for cord, initiate breastfeeding soon after birth, have the baby wiped and weighed and have trained personnel for postnatal checks. Women in the intervention group were more likely to rate their delivery as good or very good.
The authors reported EBF rates of 2% and 3% in the pre-intervention and comparison groups respectively, and 55% in the MICYN intervention and control group. The OR was slightly higher for the intervention than the control group at 2, 4 and 6 months.
The authors report that significantly more women in the intervention arm are visited by a CHW than in the control arm. More women who were helped by TBAs were visited by a CHW after birth.<br>The proportion of babies breastfed within an hour was higher in the intervention than in the control, as was the proportion of babies who were exclusively breastfed.<br>The intervention arm was better for: immediate STS care, clean cord, thermal care, KMC for LBW babies, and seeking extra care for LBWs.<br>All babies with one danger sign were taken to private providers, usually a drug shop.<br>LBW babies didn't end up getting extra visits.
90% of families had one visit in the first 10 days after birth, about 74% were visited twice and about 40% were visited the recommended 3 times. Nearly half the women had attended a women's group meeting. More neonates with severe illness were taken to an appropriate healthcare provider in the intervention than in the control. Infant mortality rate was significantly lower in the itnervention group, as was neonatal mortality after the first dat of life. NMR was lower in when women gave birth at home as opposed to at a facility.<br>Mothers in the intervention group had improved newborn care practices compared to the control group, such as early initiation of BF, EBF, delayed bathing. Home births and facility births both had better practices in the intervention group, but the effect was larger for home births. Appropriate clothing was universal in the groups, and STS contact was low in both groups.

Low rates of attendance for delivery. About half received their first visit on the day of birth or the day after. Median number of CHW assessments for neonates was 4. Referrals were suggested for 4% of the sample, compliance was a little over 50%. Women who had higher socioeconomic status, were younger, primigravida were less likely to be visited by CHWs (likely because they delivered in their parental home). Assessment rates were also lower for pre-term babies than term babies. Referrals for young neonates (less than 6 days old) were less likely to be complied with than referrals for older neonates. Referrals were more likely to be complied with for serious illness and for male babies. NMR was higher in the group that had never been assessed than the group assessed by CHWs at least once. CHWs identified and classified serious illness with high levels of sensitivity and specificity.
Compliance with referrals and independent care seeking.<br>Nearly 70% of mothers had a postnatal visit from a CBSV and agreement was very high between CBSVs, supervisors and the clinician.<br>Compliance with referrals was high (86%). Those who didn't comply thought the baby would get better by itself or had previous experience with kids and ignored advice. Most went to hospitals, clinics or facilities. Clinics would often send babies home without treatment, which resulted in a few deaths.<br>Care seeking was similar for referrals and self-recognition of symptoms.<br>All cause NMR and NMR post day 1 were reduced.
Maternal, perinatal, neonatal and infant mortality rates measured for womens intervention groups; IMR and EBF for the volunteer counselling group.<br>Women's group interventions reduced MMR, PMR and NMR in years 2 and 3. Volunteer groups reduced IMR overall infant morbidity. EBF rates increased in areas with both interventions. Coverage for both interventions was between 50-60%. Volunteer peer counsellors led to better recognition of danger signs and timely seeking of care.<br>Peer counselling resulted in higher levels of PNC and EBF, and lower frequencies of percevied maternal and neonatal issues, but newborn care practices (early wrapping and early initiation of breastfeeding) were lower; the opposite was true for the women's group clusters. Clusters with the women's group only had lowest uptake of PNC.<br>The women's group intervention cost $114 per YLL (year of life lost) averted; volunteer peer counselling cost $33 per YLL averted.

30% reduction in neonatal mortality in intervention areas. Better ANC and hygiene processes. The groups learned, developed confidence, disseminated information within communities and increased community capacity to take action. Trust in good NGOs, such as MIRA, which created the women's groups. Deep mistrust of government health facilities and providers, people felt they are not treated well. The intervention areas become more aware of their local CHWs, but little evidence that health services improved as a result. Women's groups were a source of support, people could share ideas and experiences and learn. Facilitators also felt they had learned. Participants become confident, expressed themselves openly and without shame. Some evidence of standing up to norms, e.g., inter-caste marriage. Women spread information in the community to non-group members, which improved hygiene and immunisation. Interventions included: stretcher schemes, revolving funds for emergencies, making and distributing clean delivery kits, using picture card games to discuss causes, prevention and treatment of maternal and newborn problems. Community capacity improved - increased communication between groups in the community. The kits were produced within the community and well received, and the fun helped people deal with financial strains (an issue which was commonly brought up).
Women's membership in self-help groups and those with insurance increased. Mother's knowledge on newborn danger signs also increased, there was a massive increase in mothers recognising difficulty breathing as a danger sign. The number of institutional deliveries aslo increased. The proportion of women who took no action or used home remedies for danger signs also decreased. The proportion of mothers seeking government care services decreaed, but the proportion seeking private care increased - this was due to lack of faith in government services, unavailability of doctors, poor care by doctors and nurses and lack of medicines and equipment.
Women in the education group were more likely than those in the control group to initiate breastfeeding within the first 2 hours after the birth, to still be breastfeeding at the time of the interview, and to do EBF 2.5-3 months post-discharge. The difference in early initiation of BF was due to caesarians, with those in the education group being more likely to BF than those in the control. Education group members were also more likely to bring their baby for a check within the first 7 days of birth.

Users of the new wrap on average practiced KMC for longer overall than traditional wraps. Mothers practices KMC for nearly 24 hours while in the facility, the hours of KMC decreased gradually at home for both groups, but the decline was steeper for the traditional group. 59 mothers attendd the postnatal visits, 58 reported practising KMC at home and and 55 maintained EBF until 28 days postpartum.<br>Before the intervention HCWs understanding of KMC and its benefits was limited to STS contact and treatment of hypothermia. After the intervention HCWs realised KMC was a standard method of care for LBW newborns, and which could help maintain the newborns temperature, reduce infection, promtoe breastfeeding, and increase weight gain. HCWs liked the new wrap as it could be used without assistance, was more comfortable for the baby, made breatfeeding easy. Men were more engaged with KMC using the new wrap, the traditional wrap was considered feminine. Mothers preferred the new wrap as it was easier to use in summer due to the heat.<br>Mothers said KMC reduced costs and promoted the health of the newborn, some mothers expressed happiness at being able to provide more care for the baby. All mothers said the new wrap was better; those who used the old wrap were not able to continue with household chores.
NMR and IMR decreased significantly during the intervention period.<br>98% of referred newborns survived.
Coverage of antenatal visits in both interventions group were above 60%. Significant improvements were seen with targeted newborn-care practices, including wiping the whole body of the infant immediately after delivery, deferment of bathing until after the fi rst 24 h, initiation of skin-to-skin care within 24 h, and covering the baby after birth and during massage. Commencement of early BF was higher in the intervention groups than in the control. Care seeking from unqualified medical practitioners decreased in the intervention group. NMR reduced in both intervention groups by over 50%, with a bigger reduction in the group without the crystal.
The number of home visits increased from about 5% to 12%. NMR remained the same but both mothers and HCWs thought early detection led to lives of newborns and mothers being saved.<br>Women felt encouraged to breastfeed and learned about the benefits of breastfeeding. Home visitors felt better equiped with knowledge and skills to promote breastfeeding, and to deal with false beliefs.<br>EBF rates increased while the program ran, though they had been increasing previously anyway.<br>Home visitors learned about cultural practices and norms which were harmful and counselled the mothers, which mothers said was informative and changed their habits.<br>Some women wanted their husband's involved in care, others didn't. Similar for the fathers, some were invovled and supportive and others said it was not their place to be involved.

Reduction in NMR and moderate maternal depression. Safe delivery practices increased (hand washing, clean cord care, use of safe delivery kits). The interventions were locally accepted, used participatory approaches to develop knowledge, skills and "critical consciousness", community involvement beyond the groups occured.<br>The intervention was successful because facilitators were local, locally appropriate discussion materials were used, and timing and content of meetings were flexible.<br>Criteria for selection of facilitators were chosen after FGDs with elders, women in the community, opinion leaders and headmen. Selected facilitators were trained for 5 days on listening and communication skills and the first phases of the intervetion. The second training session was 2 days long and took place after 6 months - they were trained on the process of developing stories about the cause and effect of health issues using pictorial representations. Training used a variety of methods (role play, group discussion, participatory exercises). Some activities were adapted from other similar studies, such as "But why" game, using picture cards Fortnightly meetings were held with senior team members and weaker facilitators were paired with peers to increase confidence, and given more attention.<br>Facilitators thought locally apprioriate picture cards, stories and participatory activities increased acceptability and helped with learning and planning within groups.<br>The intervention had better coverage which could have yielded better results.
Birth in a facility is the primary outcome. 1/3 of calls were about newborn illness, the most common symptoms were fever, pneumonia and convulsions<br>Health facility delivery was 3 times higher in intervention than control.<br>Drying soon after delivery was the same in both groups. Equal numbers received BCG and about the same numbers were breastfeeding.<br>Cord care, thermal care and timely care-seeking for newborns was higher in the intervention group.<br>Pre-lacteal feeds and early bathing remained an issue in the control group.
Knowledge on wiping and cutting the cord was low in both groups, but slightly higher in the control group. In the intervention, knowledge on initiation of BFwithin an hour, colostrum feeding, duration of EBF, time of complementary food initiation, bathing of newborn after 3 days and shaving of hair after one month were higher. Most members of intervention and control could name 1-2 danger signs, knowing more danger signs was higher in the intervention than control. Joint decision making for FP, ANC, postnatal care was lower in the control group. Joint decision making improved in the transitional areas, but other indicators were similar to the control areas.
Early bathing and late initiation of BF was reduced in both areas. Knowledge on newborn care practices increased more in the intervention group. Perinatal mortality decreased in the intervention area. Decreased deaths due to asphyxia and infections in the intervention area. Knowledge of danger signs improved in the intervention group.

Intervention areas had better immediate cord care, delayed bathing and EBF in intervention areas compared to controls. Respondent's knowledge on PNC was significantly higher in intervention than in control regions. Home deliveries decreased, and deliveries in public facilities increased in the intervention area. Intervention areas had significantly higher visits within 2 days of delivery than control.
There were increased visits from female CHWs and increased dissemination of information. Service utilisation also increased, including for visits after noticing danger signs. The time lag between noticing symptoms and going to the hospital decreased by 0.5 days. NMR decreased. Behaviours also changed, including delayed bathing (which was thought to be too hard a behaviour to alter), and early BF.
NMR was the primary outcome measured.<br>NMR was lower in the intervention group than the control group for each year of the study, but the results weren't significant. There were no differences in most home care practices or healthcare seeking practices between groups. Higher frequencies of delayed bathing and EBF in the intervention group.<br>The 3 main interventions implemented by the groups were creating emergency funds managed by the group, raising awareness of maternal and child health issues during meetings and in the community using materials provided by the group (cards and flipcharts), fostering effective communication with healthcare providers.<br>Authors think the intervention wasn't successful as there wasn't enough coverage i.e., too few groups per the population, too few facilitators (each had too many groups), contextual factors - floods and sociocultural norms against women joining groups/seeking care.
By 3 months the itnervention group had received more home visits by HCWs, been weighed and received immunisation visits. Mothers were more frequently counselled by health and nutrition workers in the intervention group. Mothers in the intervention received more EBF counselling at home visits, imunisation sessions and weighing sessions than those in the control. EBF rates at 3 months were higher in the intervention, and the rate of prelacteal feeds was lower. EBF for LBW babies was much higher in intervention than control. EBF was done for longer in intervention than control, same for LBW babies. At 3 and 6 months rates of diarrhoea were lower in intervention than control. There were no differences in weight or length between the groups.
Most postnatal visits were completed. EBF increased in hospital settings, but dropped off rapidly when mothers went home - at 10 days only a third were still practicing EBF. The group receiving home visits maintained EBF for longer, though it did drop with time. The BFHI alone benefitted better-off and better educated mothers to EBF, whereas the home visits all socioeconomic groups benefitted. Few infants in the home visit group were given othe liquids or food in the first 6 months.

EBF rates in the intervention clusters at 12 weeks were twice those in the control. The rate of EBF at 24 weeks dropped for both intervention and control, but the intervention group was still doing better. The rates of diarrhoea at 12 and 24 weeks weren't much different for intervention or control.
Coverage of the home visits was good, the lowest was the firt post natal visit at 63%. Group A received early visits, Group B received late visits. Group B had more mothers classed with feeding difficulties and more mothers who had each of the 6 factors in the assessment of BF problems. Not having an early CHW visit and giving pre-lacteal feeds were both associated with BF problems late in the first week.
Most NMR occurred on the first day of life, and measurement of birthweight in both groups was low. Most women in the intervention group reported ever giving CKMC, rates were next to 0 in the control group. However, about a quarter in the intervention group received KMC for more than 7 hours per day in the first 2 days. KMC was lower in hotter months, and higher for mothers who had delivered at home. BF immediately after birth and washing with damp cloths was higher in the intervention group. There was no difference between the groups for morbidity or growth at 30 and 45 days. NMR was the same between groups. NMR was lower for LBW babies n the intervention group. Diarrhoea was higher for babies in the intervention group.
Mothers across the groups did not know how long to breastfeed, or the benefits of breastfeeding LBW infants. The proportion of mothers breastfeeding exclusively from 2wks to 6mo was significantly higher in the breastfeeding counselled group than the other groups, it was higher at each time point that was examined. Women in the breastfeeding group were 6.3 times more likely to EBF than mothers in other groups. There was no difference in EBF between the control and childcare counselling group. The breastfeeding group had no incidence of diarrhoea, while the other groups experienced it. Mothers were happy with the program and in the breastfeeding group said the peer counsellor influenced their feeding decision the most.
Women in the intervention district received more antenatal and postnatal visits compared to the control group. Women who received a postnatal visit within 28 days had a lower NMR than women who received no visit or only an antenatal visit. Women who received a visit within 3 days of delivery had a 25% lower mortality than those who received no visit. Those practicing clean cord care and coverage of skilled birth attendance also increased in the interventon group.

At 3 months fathers in the intervention group had better behaviours - BF within one hour, making decisions about infant nutrition with their wife, EBF. Men in this group were also more likely to be supportive - housework, baby care, female support.<br>Some mothers also attended an education group, EBF was highest amongst those where both were educated, then where fathers were educated and lowest in the group with neither attending a session.<br>Results at 9 months were similar to the 3 month patterns - EBF is higher as is caring for the baby (diaper changes, dressing the baby).<br>Wives said men shared what they learned with them and with the community, and recommended the course (despite ridicule from other men) which lead to increased demand. They also said the education helped the couple resist social norms, such as feeding the baby water or tea before 6 months. On the downside, husbands could become know it alls.
Coverage was higher than other interventions, and just over 1/3 of women who gave birth said they were part of a group (compared to 3% prior to scale-up in the intervention). NMR was lower in the intervention group than clusters. Hygienic home delivery practices, thermal care of the newborn and feeding practices all improved. NMR was usually caused by asphyxia, infections and complications with prematurity and LBW. Infections and pre-term/small babies decreased in the intervention group but not the control. Health service utilisation did not increase much in the intervention group.
The authors rated 20% of the home visits were rated as having a performance less than expected.<br>Groups A and B had higher EBF rates than those in the control.<br>Women highly valued the home visits - could be meeting perceived needs.
Most villages in the intervention group established CHCs, half of which created emergency transport funds. Coverage for births and postnatal care was low, with no LHW covering all 4 postnatal visits. There were more visits by LHWs during pregnancy in the intervention group than in the control. NMR was lower in the intervention than control. Women in the intervention cluster were more likely to give colostrum, BF within 30 minutes of birth, delayed bathing and receiving a postnatal LHW visit. There were no differences in the prevalence of illnesses and careseeking behaviours between the groups.
Increased number of deliveries within facilities (government hospitals/health centres). The number of women receiving a postnatal visit doubled, but overall remained low (23.4%), half these checks were with HEWs. Postnatal checks with HDA members remained low. Counselling on SSC, care for LBW babies, expression of breastmilk, cup feeding and newborn danger signs all increased, but overall remained low. HEWs were better at counselling than more skilled providers.<br>SSC and EBF increased, including reduced discarding of colustrum.

Quality of counselling, facility-based newborn care practices and maternal knowledge were all measured. Immediate newborn care (thermal protection and immediate breastfeeding), danger signs, healthy home practices and FP were assessed.<br>In the intervention site mothers received more messages about recommended newborn care (cord care, STS, breastfeeding, delayed bathing).<br>Counseling on danger signs and home-based care also increased. No sig improvement on neonatal care-seeking for complications. Knowledge on neonatal danger signs and healthy home practices improved.
High levels of home visits in the control and intervention group, mostly by volunteers. The number of referrals to health posts increased in intervention areas compared to control, and HEWs mostly initiated treatment on antibiotics. Some referralsto health facilities were made, but few followed up. HEWs got mostly the same results as POs for danger sign recognition. Completion rate on antibiotics was high (over 79%). Post day 1 mortality reduced in the intervention arm, but was not significant. The % of babies treated is about half of what is expected using global estimates - so not all barriers to access care are addressed.
HIV-free infant survival and levels of EBF and appropriate feeding were recorded.<br>Initiation of breastfeeding within an hour of birth was low for both groups; at 12 weeks the intervention doubled EBF and exclusive formaule feeding compared to the control; EBF rates were higher amongst HIV negative mothers, the reduced risk of mixed formula feeding was higher in the non-HIV mothers. No effect based on household asset score or education status.
Success was measured by detecting issues with newborns, adherence to referrals and knowledge of neonatal care.<br>There was no effect on the information of neonatal care; the intervention resulted in care being sought earlier, and better recognition of neonatal complications (according to the author).
87-95% of mothers who received a referral followed up (depending on if 10 who denied receiving a referral are included). 40% were referred at 2-4 weeks. Breathing difficulties, rash and redness around the cord were the most common reasona for referral.<br>Half the mothers recognised a danger sign (breathing difficulties and fever) and took the baby for a referral, none of the mothers who did not recognise a danger sign took their babies in.<br>Median time between referral and going was 1 day, delays were usually because of not recognising how serious the issue is or the clinic being closed. Home treatment (OTC) was often given.<br>Mothers rated the QoC of CHWs very highly; mothers did not experience cost as an issue for transport.<br>Only 16% of HCWs gave written feedback to the CHWs who referred.

Attendance at ANC, facility delivery, PNC, immunisation and referrals to facilities. The authors reported an increase in ANC attendance, a slight increase in immunisation and similar results for other outcomes. In the interventions group there was a higher number of PNC visits.

**Opportunities and challenges**
**NASSS domain 6**
KMC can reduce stress for mothers of pre-term babies.
Skin to skin contact isn't unformly applied in previous literature. Breast crawl resulted in better regulated temperatures of babies - it's a method can be used in low resoruce settings.
Most births occur at home given Nepal's geography - it's difficult for women to make it to the clinics.
High NMR, unwillingness to seek treatment outside of the home

About a fifth of births occur at home.<br>Most women receive 3 or more ANC visits (per government guidelines).<br>4 facilitators left (job offers or pregnnacy), but were replaced by 3 women who shadowed them for a month before taking over.
Most women give birth at home with help from a TBA.<br>High acceptance of interventions delivered by VHWs.
Low access to electricity and sanitation facilities, over half the women in the selected areas are literate.<br>Only 10% of pregnant women in the area were members of the group.<br>Mothers are increasingly delivering at facilities - this is good, but can overwhelm the system as it is not prepared for these higher numbers.

Mothers usually only have a few years of education.<br>Most births occur in facilities.<br>Women stay at home for 4-6 weeks after delivery and don't do chores - increased the duration and number of days of STS care.<br>Belt provided to mothers to fasten children while providing KMC, in care required.	
Many neonatal death occur at home, particularly in rural regions where there are fewer skilled providers.<br>LHWs were established in the 1990s so have been around for a while.	
Breastfeeding rates had been steadily increasing in Brazil but stagnated around 2006.	
Areas with high NMR and high incidence of home delivery were selected.<br>Lack of skilled attendants at birth and low rates of facility delivery.	

High prevalence of HIV in SA.<br>TB is a major issue too.<br>Most mothers are unemployed and single.
Shortage of human resources for health, public HC facilities have poor performance, inadequate drug supplies in public facilities, Low socioeconomic status, women lack decision-making power.<br>Home deliveries are common.<br>Community clinics are used in Bangladesh - considered PPP, as the land is donated by the community and it is managed by a community group. 3 Community Support Groups are set up in each CC, with members from various groups (housewives, elderly, people with diability etc.)
High rates of institutional deliveries.
Most deliveries occur at home, hospitals may not have specialised NBUs that measure birth weight.<br>High neonatal mortality rates in the area. Transport to the facilities is difficult - infrequent and expensive.<br>Men have final say in the health of the child and the woman.
Difficult to gauge symptoms with a neonate, and morbidities have overlapping symptoms.<br>It is a common cultural practice for women to go to their parent's home when delivering, espeically if it is their first child.<br>Women often stay at home with the baby for the first 40 days.<br>Unqualified or traditional practitioners are often consulted.
HCWs and VHTs were happy - better connections between the 2 groups.<br>VHTs experiences some issues - interfering with work, no lights at night, inconsistent drug supply. VHTs also wanted to be able to treat newborns.<br>Carers appreciate the service of VHTs and give them lifts and money.

NHC (neighbourhood health committee) links CHWs and TBAs with the formal health system - community based health management structure. Represent communities on health centre committees.<br>Rural undeveloped district; Lacks physical infrastructure.<br>CHWs were moslty male and TBAs mostly women; only about 20% had this role as their full-time role.<br>Zambian government has created a new cadre of community health assistants who will be paid monthly allowances.	
Half the women give birth at home hence the need for a community program.<br>The groups with wider community members may not meet regularly due to lack of financial incentives, however, having these groups makes the intervention inclusive - important given the patriarchal norms.<br>Mothers sometimes created IGAs from their support groups.	
EBF rates are low in most LMICs, especially at 4-6 months.<br>Breatfeeding is affected by maternal health, occupation, knowledge, education, method of delivery and breastfeeding intention.	
High ANC attendance, but low PNC attendance.<br>Differences in care received in urban and rural (urban better served).<br>Low levels of women receiving the care the government recommends.<br>One region had issues with access to facilities, therefore home births were more common.	

KMC was near 0 at baseline.<br>Ethiopia has lack of funding for training and supplies, lack of space for KMC in facilities and lack of staff assigned to support KMC.<br>India - difficulties identifying LBW babies, limited health worker skills/knowledge on KMC, lack of space in facilities for KMC.<br>Mothers and families have positive responses to initiating KMC at home.<br>25-50% of mothers were illiterate; home births were frequent in Ethiopia; some regions in India had increased use of private facilities for birth; postnatal check ins Ethiopia were low.	
The Indian government adopted a home-based newborn care strategy for improving community care practices by fostering a supportive environment via ASHAs, and aiding early identification of sick newborns for appropriate care and timely referral.<br>Studies found continuous training was required for ASHAs to retin knowledge and build communication skills.	
Well established primary healthcare system.<br>ASHAs are well established - been used since 2005 and cover ANC, postnatal care, immuisation etc.<br>Intervention areas had people with lower levels of literacy and were inhabited by tribes<br>Some areas were excluded because they did not have internet.	
EBF is still relatively low in India (about 50%), and the duration is not close to the WHO recommended 6 months.<br>BF cessation rates are high in the first month.	
Newborns leave the faciltiy early due to a shortage of beds, the perception that better care can be provided at home, financial constraints.	

The Indian government introduced MCH programs to encourage institutional delivery and care, these only came into the CHAMPION areas towards the end of the trial.<br>Area has one of the highest NMRs in the country.
Urban slums have low quality private facilities unlikely to adhere to BFHI.<br>Women need to return to work fairly soon after birth and there's lack of cultural acceptance of breast milk expression.<br>Kenya has a community health strategy based on CHWs, and a baby friendly community initiative based on BFHI.<br>Mothers reported being hapy with the frequency and content of the visits from CHWs (intervention group).
Retention of CHWs was 100% in the project.
Half the mothers are uneducated/illiterate.<br>2/3 of births occur at home.<br>Care seeking from unqualified private practitioners is common.

Mothers deliver in their parental homes.

Rural regions; low levels of education; lack modern infrasturcture; distance to facilities can be high;

Most women attend at least one ANC visit; more than half the women deliver in facilities.
Shortage of personnel, low morale and irregular drug supply.

Most births occur at home. Mothers-in-law are major decision makers. Arranged marriages are common. Maoist insurgency (2004-2006). Access to water is an issue, and most people do not own their own land.
Mother's lacked knowledge on newborn danger signs, had low social standing and lack of education.
The number of ANC visits is improving but quality varies, those in the upper and middle classes often access better care.<br>BF initiation happens early, but EBF rates are very low; often other food or liquids are introduced early.<br>ANC visits do not cover everything mothers would like.<br>Women stay at home with the baby for the first 40 days after delivery.

KMC is part of the national policy for care of LBW babies, included in training for HCWs, however KMC isn't widely practiced or promoted.
High number of institutional deliveries.<br>Mother and baby are often home within 48hrs, the next visit is at immunisation.
High NMR - one of the highest in India and the world.<br>Low socioeconomic status.<br>Low levels of care seeking - 90% of deliveries happen at home, low ANC attendace.<br>Confinement of mothers and newborns was a common practice, and overlapped with when most issues were seen.<br>Low levels of literacy in the female population.
Humanitarian setting with ongoing conflict<br>Mothers are usually discharged 6 hours after birth<br>All pregnant women receive an MCH book

The area is forested, remote and hilly - difficult to access healthcare.<br>High NMR rate.<br>80% of deliveries occur at home, with either a TBA or relative of the pregnant woman present.<br>Most people believe in supernatural beings, health issues are often attributed to supernatural causes.<br>Government implemented National Rural Health Mission t improve access to quality care. Consists of health system strengthening, ASHAs and village health committees to address local issues and monitor health services, voucher scheme for facility deliveries.<br>Coverage was variable in the study sites.<br>Interruptions during festival, harvest and migration periods.	
Mobile phone penetration is high.<br>Long distances between homes and facilities.	
Patriarchal culture, women's access to health and socio-economic institutions happens via men.<br>Women's health is often not prioritised, with morbidity during pregnancy considered normal.<br>Social stigma for men to be involved in women's health. Men get shy/embarassed when discussing these matters in public.<br>Men are focused on their work responsibilities.<br>Poverty is common, illiteracy is high.	
There is a research centre in the area that has been collecting data on marriage, births, deaths etc. for a long time. They have also run health programs targeting maternal and child health.	

Populous country. High at-home delivery rates. Unavailability of maternal services. Lack of education. Low socio-economic status. Lack of autonomy - decisions made by in-laws, husbands and traditional healers.
Lack of human resources; lack of infrastructure (transport and health); half the population is literate in both.
Most births in Bangladesh occur at home.<br>Women traditionally go to their mother's home just before giving birth.
Low rates of literacy - for both men and women.<br>Maternal undernutrition is high.<br>Open defecation is common.
A quarter of the female population is illiterate.<br>90% of women give birth at a hospital.<br>Pre-intervention data showed EBF was low, and duration of BF of any kind was short.<br>About 1/3 of mothers were adolescents, and about 1/3 were having their first baby.

In SA formula is provided by the government, partly in programs to avoid transmission of HIV, and marketing of breastmilk substitutes hasn’t been done - so mixed messages can cause issues.<br>EBF is already high in Uganda and Burkina Fasso.
Delayed BF and prelacteal feeds are common.<br>Mothers often go to their parent's homes to deliver.<br>National BF program focused on urban hospitals and training doctors and nurses, involvement of grassroots workers is absent.<br>High rates of home deliveries. Women stay at home with the baby for the first 1-2 weeks post-delivery.
Incidence of home delivery, LBW and neonatal and infant mortality is high and neonatal intensive care is unavailable.<br>Lack of skilled attendance at birth - weights often not measured.<br>Non-family members often not allowed in the birthing area for the first few days after delivery.
Breastfeeding for 6 months also allows for birth spacing - can break intergenerational loops of LBW babies.<br>Mothers don't receive information about EBF when they are discharged from the hospital.<br>Mothers were happy with the program.<br>Only half the counsellors were willing to do home visits.
Rural area.<br>Only a quarter of mothers received a postnatal visit within 3 days of delivery.

Large workplaces (over 1000 people) are required to have physicians attached to them for employee health education.<br>Low rate of EBF at 6 months.<br>Parents receive ANC, but no information about what happens after delivery. Parents know what info they would like to have.
One third of members were men.
Women do not have decision-making authority. High rates of illiteracy.<br>The hospital has a policy of separating newborns and their mothers after delivery. Short stays in hospital post delivery.<br>Societal preference for large families.<br>Home visits are part of a national government program but are not implemented due to lack of community nurses (a new cadre of workers)<br>Women rely on BF as a form of contraception post delivery.
Rural areas have higher NMRs than urban areas.<br>65% of births take place at home, half of those are with untrained TBAs who charge for their services.<br>LHWs were introduced in 1994 - well established. Do home visits or wfh (health homes).<br>Low literacy levels for women. Low socioeconomic status.<br>Mothers give birth at their parents house and don't have contact with anyone for 40 days.
Most mothers don't give birth in facilities (only 16% do)<br>KMC being expanded in facilities - not all offer it at the moment, but HCWs in the area were trained - may have lead to the success seen with facility births.<br>HEWs have multiple roles, sometimes they aren't at the facilities and the facilities are far from the mothers. Other programs have had higher coverage when using HEWs - but they have more resources.

Whether women give birth in facilities or at home postnatal services are often inadequate, especially around information sharing. Pictorial job aids have proven effectvive when used by HCWs for ANC. Delivery rates on public facilities are high in Benin, and most women are attended by a skilled birth attendant in the country. Health worker strikes occured - fewer births in facilities as a result. HCWs problem-solved with mothers so that suggestions for home care could actually be implemented.	
Most women in Ethiopia can't access care in facilities. Referrals decreased towards the end of the study as volunteers knew the project was wrapping up, and the government introduced HDAs. Cultural practice of keeping newborns at home, keeping babies away from strangers and the sun.	
SA has the lowest rate of EBF in SSA at 6%. High HIV prevelance; low employment rate; high infant mortality rate; most mothers are single. High levels of mobile phone penetration. Other people- mothers, friends and boyfriends need to be included in breastfeeding decision making (based on qual sub studies). Lack of breastfeeding initiation within an hour of birth in all groups - this can reduce 22% of neonatal deaths.	
Urban poor group - less likely to come back for checkups due to financial and time constraints. Urban poor groups have worse outcomes for neonatal health. Smartphone penetration is high, allowing for mhealth apps to be used.	
High HIV prevalence; delayed care-seeking for sick children; primary health centres revitalised + integrated management of childhood illness introduced; most mothers are young (24), single and unemployed; facilities are easy to access (walking distance); CHWs were carefully selected and respected within the community; referral letters reduced waiting times for mothers;	

The population won't include women who give birth at home, who are arguably most at risk.

Contir	
**Informational**	**Management**
**Use of information being shared and used appropriately**	**Management of an illness in a consistent and coherent manner.**
Info about KMC given to mothers - unclear how this donw, how often or if there were any handouts given.	
Information is passed on to mothers to look out for danger signs, and information is passed to CHWs on signs to look out for and how to treat mild infections or refer for further care.	The algorithm used to diagnose, treat and refer newborns helps CHWs to manage severe illness in a consistent manner.

Information is shared between the VHWs and the mothers via the education sessions and support (e.g., with breastfeeding and KMC).	
Health education passes on information to mothers.	Training means that HCWs will manage conditions according to protocols e.g, for haemorrhage or asphyxia.

Mothers are provided information on KMC and its importance for LBW babies during the visits from study staff.	
Information is shared with LHWs and it is expected that they will share this information with mothers to improve neonatal outcomes.	LHWs are given training on when to administer antibiotics and resuscitate babies. This standardised response may have lead to improved outcomes.
Information about EBF is shared via the booklet and the online group.	
Information was passed on from CHWs to mothers on newborn care practices, using charts, DVDs and verbal communication.	

Information on best care practices (feeding, keeping the baby warm via KMC, danger signs) were shared with the mothers by the CHWs.	
The community workers shared information on birth planning, where to seek care and when.	
HCWs were trained on ENC and in turn disseminated this information to mothers before they were discharged. This lead to changes in harmful practices around cord care.	
Information is shared with mothers via CNPs and visual flyers, which lead to uptake of KMC.	
Mothers and family members were informed about 13 danger signs, using pictorial aids, and were told to seek care if they saw any of the danger signs.	Families were told to seek care for any of the 13 danger signs they were taught.
Information on danger signs and referrals to care were provided.	The VHTs all received the same training and provided referrals to facilities rather than treating the babies themselves - standard care.

Information is being shared by the ToTs to CHVs, who then share it with the community. Repeated home visits and provision of educational material helps get the message across.	
Information on the benefits of breastfeeding and how to support wives who are breastfeeding is shared with fathers using a variety of platforms - group counselling, individual counselling, health messages on loudspeakers, posters etc.	
	Mothers staying for 24hrs or receiving a home visit 48hrs after birth was made consistent.

Interventions focused on information sharing - HCWs were upskilled, information passed to mothers, helplines exist.	
ASHA's provide information on newborn care to mothers and family members, and faciltiate discussions in group settings in the community.	
The videos shared with families were about health messages, which proved to be effective.	
Information was shared with the mothers about correct breastfeeding technique if they has low LATCH scores before discharge. The demonstrations provided support for mothers.	
	The videos and calls with nurses ensure that the babies are receiving the right care, at the right time and in a consistent manner.

Information was shared with the mothers on danger signs and when to seek care, and the mothers awareness of local service provision was improved.	
CHWs provided education and advice constantly, via home visits or educational materials.	
CHWs were sharing information with mothers on best practices for newborns.	
Information about newborn care practices are shared with mothers during home visits.	HCWs across the cadres are given training on managing newborn illnesses.

Informational continuity was improved via use of the cards, which mothers took to each visits. Mothers were also taught about newborn care when CHWs visited them antenatally.	An algorithm was used to aid identification of illness for the CHWs.
Information on danger signs shared with mothers.	
Information about breastfeeding, danger signs and referral to care provided by the peer counsellor.	

Information from the groups is shared with the wider community by group members - the whole community received this information well and appreciated it.	
Information is shared with pregnant women in the community, using education material such as flipbooks, to improve awareness of danger signs and care seeking.	
Information about newborn care is given to mothers during the session. The women may have felt supported by receiving that information.	

Information about KMC is shared with mothers from trained HCWs at the hospital.	Managing LBWs with KMC became consistent, but the ease of KMC was improved with the new wrap, e.g., fathers were able to participate and mothers could continue with household chores while using the new wrpa.
CHWs advised mothers on KMC and BF.	
Information on essential newborn care was shared with the community in a variety of ways - home visits, folk songs etc.	

VHTs are sharing information from their training and providing advice from HCWs where required.	
The existing program aimed to include men in decision-making, building their knowledge on issues such as ANC and postnatal care.	
Information on danger signs is shared with mothers during CHW visits to their homes.	Checklists and the algorithm used by CHWs ensures that management of illnesses is consistent.

Information on newborn care practices are shared with mothers via CHWs. This lead to improved cord care and EBF.	
The female CHVs share information with mothers and family members on danger signs and care rpactices after delivery, as well as antenatal practices and maternal health.	
Information on maternal and child health shared between the groups and the community.	
Information on EBF and its benefits are passed on to mothers by various HCWs. Messaging around continuing EBF for 6 months were well receieved.	
Information on EBF and its benefits are shared with mothers, both in the hospital setting and in home visits. Booklets were used as discussion tools during the home visits.	

Information about breastfeeding is shared with the mothers from the trained peer counsellors (training based on WHO guidelines on breastfeeding and HIV).	
Information is shared with the mothers via CHWs about BF, including positioning, attachment and sucking.	
Information on KMC, BF early and delayed birthing is shared with mothers.	
Information is being shared between the peer counsellors and the mothers on EBF and its benefits.	
Information is shared with the community workers who then go and share it with the mothers.	

Information is shared with fathers on healthy behaviours and communication, which they then implemented with their partners.	
The midwife shares educational messages with the mothers, messages received during the 5 day training.	
Information was shared in village community groups with women, and other members of their families.	
HCWs were trained on SSC and EBF and passed the information on to mothers during ANC and PNC visits.	

Information is being shared between the HCWs and the mothers via reminders from the pictorial job aids.	The checklist provides standard care to all mothers.
	Danger signs were taught and recognition of one danger sign meant babies could receive antibiotics, or be referred to higher level facilities if required.
Information is being shared between the CHWs and the mothers to improve outcomes.	
Knowledge about danger signs for mothers and neonates is shared with the mothers, as if information on whetehr referrals are necessary.	The checklist provides a standard of care - referrals are provided in a consistent manner?
Information is being shared between the CHWs and the mothers to improve outcomes.	Referrals were provided for danger signs that were recognised by the CHWs.

nuity Logic	
Relational	Interpersonal
Ongoing relationship between a patient and one or more providers	Better interpersonal relatisonsips (family or between HCWs)
	Encouraging breastfeeding early on improves breatfeeding post-discharge by improving maternal bonding with the child.
Repeat visits build a relationships between the mothers and the health system. Encourages referal for sick babies, and treatment for babies.	
	The group meetings in the community may improve relationships within the community by discussing the need for maternal and neonatal care seeking. The male mobilisers may be particularly helpful in getting other men onboard with the intervention.

	It took time to form relationships between the facilitator and group, but once a good relationship was built it really aided discussions.	
The VHWs make multiple visits, but it's unlcear if this builds a solid relationships between them and the mothers. However, mothers do accept their services, so probably appreciate the support/advice they receive from the VHWs.		
	Authors say the intervention leads to better networks and solidarity.	

Multiple visits ensure women get support while implementing KMC and breastfeeding.		
	The authors reported better relationships between TBA and LHWs.	
Relationships are built between the mothers in the group and the moderators, who encourage discussion and respond to their questions. The group provides support.	Discussions in the group probably build relationships between mothers in the intervention group.	
The repeated visits may have build a relationships between the mother and the CHW.	The intervention also consisted of community mobilisation, which may have increased awareness of issues relating to newborn mortality.	

The visits provided a link between the mothers to the facilities post-discharge.	
	The discussions and visits from community workers may have changed the views of the community regarding the continuum of care.
The multiple visits from the CNP before and after delivery may have built up trust. One father was hesitant about KMC but after learning about it from the CNP was happy to help with the process.	
Relationships between the carers and VHTs were built, the community appreciated them and listened to their advice.	The relationships between the VHTs and HCWs was also improved - with both appreciating the other.

The intervention made it more likely for women to receive visits - key for the 6-8 week postnatal checkup.	Better relationship and communication between TBAs and CHWs - task sharing, communication, trust.	
	The intervention focuses on fostering a supportive relationship between husbands and wives regarding breastfeeding. The competition held within the community also encourages social change around BF and encourages partners to become involved.	
The number of contacts between mothers and health facilities increased with the intervention.		

Home visits by CHWs to support KMC continuity.	Community events and use of champions leverages social connections to improve care.	
ASHAs built rapport with the family members, and presumably the mothers, during their multiple home visits.	The community meetings and rapport building with family members may have created better relationships within the community and in households.	
The schedules and apps encouraged more contact between the ASHAs and the mothers in the community.		

The CHWs seem to build a good relationship with repeat visits, for example, mothers defer to them for advice on when to return to work.	

Relationships between the community-based CSBVs and the mothers helped improve compliance with referrals.	
	The iterative cycle probably built relationships between the women and the group facilitator

	Better relationships are built within the community - people speak to each other more, and the group provides support to mothers.	
	The villae community groups may have lead to better relationships within the community, especially since there was a focus on gender equity.	

Home visits were made after babies were discharged from the hospital, to provide more regular follow-up.	The HBNC coordinator was the link between the CHWs in thw community and HCWs at the facility. This middle role allowed greater coordination between the different cadres.
The repeated visits lead to relationship building between the community volunteers and the mothers. This lead to more seeking of formal care from mothers in the intervention groups.	
HCWs said the visits made them more empathetic and human, it built the relationships with the mothers. The women felt valued and cared for.	

	Relationships are built within the community, critical consciousness was developed. Group members would regulalry help out non-members too.
VHTs gained status as those they referred for care received prompt treatment - promoted community confidence in the health system and pormotion of maternal and newborn care.	
	The existing program aimed to include men in decision-making, creating better joint decision making dynamics within families. The group discussions among men may have also provided support and encouraged involvement of men in decision-making.

Unclear if the CHWs built relationships with the mothers they visited.		
CHWs are respected members of the community and are seen as credible and trusworthy sources of health information.		
Imrpoved referrals could have lead to better patient provider relationships, as could the training on communicaiton.	The groups also interacted with the community to educate them - improves links.	
	Home visits by CHWs reached family members, who are important stakeholders for infant feeding practices.	
Mothers received multiple postnatal visits from CHWs, this would have built their relationship and created trust.	CHWs checked to see whether other family members were supportive of BEF and encouraged them to help with household chores.	

Unclear if strong relationships are built between the peer counsellors and mothers, but the mothers can receive referrals to care if they are struggling which may help.	
CHWs make multiple visits and provide support to mothers who may be experiencing issues with BF.	
The peer counsellors built a relationship with the mothers and gave them information and support while practising EBF. The peer counsellors influenced feeding decisions the most.	
The community workers are meant to make multiple visits, potentially building a relationship with the mothers. Visits at ANC may build these relationships to make postnatal visits easier.	

	Authors don't explicitly say, but relationships were probably built within the groups, which aided seeking care.
The midwife provides emotional support during the home visits and is able to answer any questions that the mother may have.	
The multiple visits from LHWs may have built a better relationship between mothers and LHWs, and the wider health system - this was demonstrated with increased numbers of deliveries in facilities in the intervention group.	

Multiple visits at home, but doesn't mention a relationships forming between mothers and the CHWs.	
The home visits/calls provide a postnatal check-up and contact with the health service. This can be especially important soon after delivery when mothers have a lot of stress/work, and seeking care in a timely fashion can reduce adverse events from things such as sepsis.	
CHWs made repeated visits and mothers trusted their advice.	

Nurses are encouraged to see mothers through the continuum of care as they get financial rewards		

Participatory Methods
**Approaches + Methods**
**Is the participation in name only?**
Not participatory.
Not participatory.
Not participatory.
Recall bias for behaviours.<br>CHWs did not attend many births because of their workload and large travel distances.<br>There was some mixing of interventions as people discussed delayed bathing in mosques.

The groups consisted of local stakeholders and practitioners, which helped contextualise the work. Each group agreed on what the problems were and came up with their own solutions. The facilitator just helped with the process. Plan-do-study-act and brainstorming were popular methods that were used by the facilitators.
N/A
The authors talk about plan, study, do, act cycles, unclear how it was ensured that the activities were participatory and who led/guided these activities.

N/A
N/A
A multidisciplinary team drafted the booklet for 6 months. 24 topics were included, and the team was trained to advice mothers on these matters. A graphic designer created the booklet.
N/A

N/A
Community support groups organised meetings and developed action plans in a participatory manner, with help from facilitators. CSGs are part of a government program that is meant to enhance use of community centre's.
N/A
N/A
N/A
N/A

N/A
N/A
N/A
N/A

Mothers, carers, community members and health providers were involved in the design and implementation.<br>Formative research informed development of the first implementation model (addressed providers' skills, community acceptance, systems components and services), this was tried and refined in rounds until the required coverage was reached. Feedback was qualitative and quant, there were 3 rounds of refinement after initial implementation, taking 5-6 months each.
N/A
N/A
N/A
N/A

N/A
N/A
A design workshop with stakeholders is mentioned - this formative work influenced the project, with health facility strengthening added to the program.
N/A

N/A
N/A
4 phase cycle used to come up with interventions (described earlier), but unclear what the intervention they came up with was.

The groups were participatory and women came up with interventions themselves, then implemented and evaluated them.
A rough draft of the health education material (pictures and health messages) were presented to mothers to ensure they made sense, changes were made before publishing the flipbook. Local dialect and local words were used.<br>A trained social worker facilitated Community's Action Experience Learning Cycle (CAELC) through VCCs to explore and collectively act upon their priority maternal and child health issues.<br>Social workers ensured participatory health education to all CBO members on newborn danger signs during their monthly village based meetings, the flipbook was used in these sessions.
N/A

N/A
N/A
Participatory social mapping of villages in the study area provided an introduction to the community, initiated the process of collaborative engagement, identified community resources for newborn health, and facilitated the planning of home visitations and group interventions. Qualitative research provided the evidence base for investigators and community members to codevelop the intervention strategy, which underwent further refinement based on findings of trials of improved practices.
N/A

The stories and games played are participatory, the facilitators are local and receive training to help them with parrticipatory communication. The stories were meant to help change behaviour by making people aware of the link between causes and effects. This creates "critical consciousness" - individuals and groups become aware of the oppressive systems and actors that maintain poverty and ill health.<br>The groups were open for anyone to attend, including men and government workers. Women shared their problems in village and cluster level meetings. Community members supported the implementation of the groups' strategies. Most group meetings had ASHAs, Anganwadi workers or auxiliary nurse midwives.
N/A
N/A
N/A

N/A
N/A
The intervention described is participatory from start to finish (problem identification to implementation and assessment). The groups could pick which interventions to implement, and the facilitators themselves were local women from the community.
N/A
N/A

N/A
N/A
N/A
N/A
N/A

N/A
Not clear - particapatory learning action cycle used.
N/A
N/A
N/A

N/A
N/A
N/A
N/A
N/A

N/A

**Opportunities, challenges and limitations**
N/A
N/A
N/A
N/A

Pregnant women from the community were not involved - so not completely participatory.
N/A
Groups came up with their own ideas and got to test them out and see what workes - buy in.<br>Allows groups to tailor interventions to their own local context.<br>Only 10% of pregnant women in the area were part of the groups.<br>Unclear how inclusive they were - were the most marginalised women allowed to participate?

N/A
N/A
Use of tech is likely to increase and should be embraced, but means replicability is limited to those with smartphones and knowledge of tech.<br>Sharing experiences allows women to not feel guilty about difficulties with EBF.
N/A

N/A
Men were involved in the CSGs, which may have helped (contribution to funds) or hindered (women may have felt uncomfortable discussing issues in front of men).
N/A
N/A
N/A
N/A

N/A
N/A
N/A

Government buy-in helped with securing funding in some regions; previous research on KMC enhanced receptivity; supportive media highlighted the work and maintained motivation; decision-makers and health managers were rewarded.	
N/A	
N/A	
N/A	
N/A	

N/A
N/A
End-users weren't consulted in the process; not enough detail about the workshop included.
N/A

N/A
N/A
Men were involved in phase 3 of the process - builds wider community buy-in.

Being inclusive - the group is open to everyone but those who are married and live closer to intervention sites participated the most.<br>The mistrust of government healthcare makes it hard to link interventions to the health services.<br>Maternal and child health is thought to be in the domain of women, hence the intervention was acceptable.	
A range of groups were involved in the intervention. Parts of the intervention were participatory, but it is not a wholly participatory intervention.	
N/A	

N/A
N/A
The intervention was based on formative researcg, which respected the views of the community (e.g., spirits causing illness). There was also trust built with the community before initaiting the intervention. Messages were designed around existing practices, cultural values and traditions so they don't seem externally enforced.
N/A

Difficult to build rapport with tribal communities initilly, participants had idea of financial gain, cancellations during festivals, had to manage the presence of men during sensitive discussions, sometimes there were conflicts within groups - in-laws and TBAs didn't want to implement certain ideas as they though they went against culture. Home-based care improved but care seeking took longer to improve.	
N/A	
N/A	
N/A	

N/A	
N/A	
Sociocultural barriers to women participating in the groups or seeking care.<br>Floods made access to the areas difficult.<br>Supervisors lived far from facilitators and didn't provide continuous support.<br>Other NGOs worked in the area and compensated women for their involvement in activities (this study didn't do that), hence women may not have wanted to be involved.<br>Few government interventions have successfully provide consistent prenatal and postnatal checks for women - participatory groups could help with this.	
N/A	
N/A	

N/A
N/A
N/A
N/A
N/A

N/A	
Religious holidays, flooding and harvest commitmetns led to some delays in the monthly meetings, but by September 2011 all groups had held 20 meetings.	
N/A	
N/A	
N/A	

N/A

N/A

N/A

N/A

N/A

N/A
